# SQLE promotes pancreatic cancer growth by attenuating ER stress and activating lipid rafts-regulated Src/PI3K/Akt signaling pathway

**DOI:** 10.1038/s41419-023-05987-7

**Published:** 2023-08-04

**Authors:** Ruiyuan Xu, Jianlu Song, Rexiati Ruze, Yuan Chen, Xinpeng Yin, Chengcheng Wang, Yupei Zhao

**Affiliations:** 1 Department of General Surgery, Peking Union Medical College Hospital, Chinese Academy of Medical Sciences, Peking Union Medical College, Beijing, 100023 P. R. China; 2 Key Laboratory of Research in Pancreatic Tumor, Chinese Academy of Medical Sciences, Beijing, 100023 P. R. China; 3 National Science and Technology Key Infrastructure on Translational Medicine in Peking Union Medical College Hospital, Beijing, 100023 P. R. China; 4 State Key Laboratory of Complex Severe and Rare Diseases, Peking Union Medical College Hospital, Chinese Academy of Medical Sciences, Peking Union Medical College, Beijing, 100023 P. R. China; 5 Medical Science Research Center, Peking Union Medical College Hospital, Chinese Academy of Medical Sciences, Peking Union Medical College, Beijing, 100023 P. R. China

**Keywords:** Cancer metabolism, Oncogenes

## Abstract

Pancreatic cancer (PC), a highly lethal malignancy, commonly exhibits metabolic reprogramming that results in therapeutic vulnerabilities. Nevertheless, the mechanisms underlying the impacts of aberrant cholesterol metabolism on PC development and progression remain elusive. In this study, we found that squalene epoxidase (SQLE) is a crucial mediator of cholesterol metabolism in PC growth. We observed a profound upregulation of SQLE in PC tissues, and its high expression was correlated with poor patient outcomes. Our functional experiments demonstrated that SQLE facilitated cell proliferation, induced cell cycle progression, and inhibited apoptosis in vitro, while promoting tumor growth in vivo. Mechanistically, SQLE was found to have a dual role. First, its inhibition led to squalene accumulation-induced endoplasmic reticulum (ER) stress and subsequent apoptosis. Second, it enhanced de novo cholesterol biosynthesis and maintained lipid raft stability, thereby activating the Src/PI3K/Akt signaling pathway. Significantly, employing SQLE inhibitors effectively suppressed PC cell proliferation and xenograft tumor growth. In summary, this study reveals SQLE as a novel oncogene that promotes PC growth by mitigating ER stress and activating lipid raft-regulated Src/PI3K/Akt signaling pathway, highlighting the potential of SQLE as a therapeutic target for PC.

## Introduction

Pancreatic cancer (PC) is a distressing malignancy that claims the highest number of fatalities and is regarded as the seventh principal cause of cancer-induced mortality globally, in both sexes [1, 2]. Moreover, it is foreseen to become the second most frequent cause of cancer-associated deaths in the United States by 2030. Surgical resection currently serves as the exclusive curative treatment for the ailment; however, the projection post-surgery is considerably bleak, with merely 20% of patients surviving up to 5 years [3]. Although several therapeutic procedures, such as chemotherapy, radiotherapy, immunotherapy, and targeted therapy have surfaced in the last few decades, the outcomes remain somewhat insubstantial. Consequently, it is imperative to explore the molecular mechanisms that lead to tumorigenesis and progression in PC, to discover novel targets and develop effective therapeutic strategies.

Metabolic reprogramming, a cancer hallmark, entails tumor cell metabolic pattern alterations to accommodate rapid proliferation [4]. Besides aerobic glycolysis and non-canonical glutamine metabolism [5, 6], aberrant lipid metabolism is a powerful contributor to the malignancy of PC [7]. Recent evidence demonstrates that the de novo fatty acid biosynthesis fosters gemcitabine resistance in PC via endoplasmic reticulum (ER) stress [8]. Cholesterol, another key lipid component, is integral to mammalian cell membrane formation and can produce steroid hormones and bile acids [9]. Abnormal cholesterol metabolism is also important for tumorigenesis and cancer progression [10]. Epidemiological findings link elevated serum cholesterol levels and dietary cholesterol intake to heightened PC risk [11, 12]. While some studies associate cholesterol-lowering medications (statins) with improved PC patient survival [13, 14], a meta-analysis negates any association between statins and decreased population-level PC risk [15]. A recent investigation elucidates this discrepancy, revealing that statin-induced cholesterol biosynthesis disruption promotes the PC basal phenotype, yielding unfavorable patient outcomes [16]. Consequently, identifying key targets related to cholesterol metabolism in PC warrants further investigation.

Squalene epoxidase (SQLE) serves as the second rate-limiting enzyme in cholesterol metabolism, by facilitating the transformation of squalene into 2,3-epoxysqualene. In recent years, abundant evidence has highlighted the overexpression of SQLE in different cancers, rendering it crucial for the malignant behavior of cells [17–20]. Since the roles of SQLE in the tumorigenesis of PC remain uncertain, this study strives to shed light on the molecular mechanisms involved in SQLE for PC tumorigenesis. Additionally, the study provides preliminary evidence for targeting SQLE as a potential therapeutic approach for PC.

## Materials and methods

### Clinical samples and tissue microarray

Paired tumor and adjacent non-cancerous pancreatic tissues were procured from patients who underwent surgery at Peking Union Medical College Hospital (PUMCH). The study was conducted after obtaining written informed consent from the patients, and it was approved by the PUMCH Ethics Committee. Furthermore, PC tissue microarray (TMA) was purchased from Shanghai Outdo Biotech (HPanA170Su04), which provided comprehensive clinical and pathological data for all samples.

### Cell culture and reagents

The PC cell lines PANC-1, MIA PaCa-2, AsPC-1, and BxPC-3, and a human normal pancreatic ductal epithelial cell line, HPNE, were obtained from the American Type Culture Collection (ATCC). Dr. G. Yang (PUMCH) generously provided the T3M4 cell line. The cell lines were cultured in DMEM or RPMI 1640 supplemented with 10% fetal bovine serum and 1% penicillin-streptomycin. All cell lines were examined regularly for mycoplasma and were maintained at 37 °C in 5% CO_2_ in a humidified environment.

Chemical compounds: terbinafine (#HY-17395A) and methyl-β-cyclodextrin (#HY-101461) from MedChemExpress; cholesterol water soluble (#HY-N0322A), LY294002 (#HY-10108), and 740 Y-P (#HY-P0175) from MedChemExpress; NB-598 (#S0338) from Selleck; and squalene (#S817864) from Macklin.

### Western blotting

Protein extraction was performed using 2% SDS lysis buffer, which had a protease and phosphatase inhibitor cocktail (#78440, Thermo Scientific™). Equal amounts of protein were subjected to SDS-PAGE, transferred to nitrocellulose filter membranes (#P-N66485, Pall), followed by an overnight incubation with specific primary antibodies at 4 °C. The membranes were visualized with SuperSignal™ West Pico PLUS Chemiluminescent Substrate (#34580, Thermo Scientific™). Supplementary Table S1 contains detailed information about the antibodies used.

### Immunohistochemical analysis

The detailed Immunohistochemical (IHC) analysis was conducted using the same methods as described previously [21]. Primary antibodies used include: anti-SQLE (#12544-1-AP, Proteintech; 1:200), anti-Ki67 (#27309-1-AP, Proteintech; 1:2500), and anti-cleaved caspase 3 (#9661, Cell Signaling Technology; 1:200).

### Lentiviral infection

Genechem (Shanghai, China) supplied lentiviruses containing short hairpin RNAs (shRNA) targeting SQLE (#109690-1, #109691-1) and overexpressing SQLE (#79811-1). We infected different cell lines with the lentiviruses following the provided manuals.

### RNA extraction and quantitative real-time PCR analysis

Total RNA extraction from samples and subsequent reverse transcription were performed using TRIzol reagent (#15596026, Life Technologies) and PrimeScript™ RT reagent Kit with gDNA Eraser (Takara, #RR047A), respectively. The expression of *SQLE* was quantified through triplicate quantitative real-time PCR (qRT-PCR) using PowerUp™ SYBR™ Green Master Mix (#A25742, Applied Biosystems™). *SQLE* expression was normalized to *GAPDH* using the 2^−ΔΔCt^ method. The primer sequences used for *SQLE* were forward 5′-CTTGACCGGTGCCACCAAAG-3′ and reverse 5′-GTTCCTTTTCTGCGCCTCCTG-3′, while those for *GAPDH* were forward 5′-GTCTCCTCTGACTTCAACAGCG-3′ and reverse 5′-ACCACCCTGTTGCTGTAGCCAA-3′.

### Cell growth assessment

For the cell growth assessment, 3 × 10^3^ PC cells were seeded in 96-well plates. At the appropriate time intervals, 10 μL of Cell Counting Kit-8 (CCK-8, #CK04, Dojindo) was added and the plates were incubated at 37 °C for 2 h. Absorbance was measured at 450 nm and 630 nm using a microplate reader.

### EdU cell proliferation assay

To assess the proliferative capacity of PC cells, the BeyoClick™ EdU Cell Proliferation Kit with Alexa Fluor 488 (#C0071, Beyotime) was utilized for the EdU cell proliferation assay following the manufacturer’s instructions (working solution 10 µM). The cells were imaged with a Nikon AX/AX R confocal microscope, and the ratio of EdU-positive cells was determined using ImageJ.

### Colony formation assay

To assess colony formation, cells were seeded into six-well plates (1000 cells per well) and incubated for 14 days. Following the incubation period, the colonies were stained with 0.1% crystal violet in methanol, and counted.

### Cell cycle assay

To perform cell cycle analysis, PC cells were harvested and washed using cold PBS. They were then fixed in 70% cold ethanol at 4 °C overnight. Subsequently, the cells were stained with propidium iodide (#C1052, Beyotime) and analyzed via flow cytometry using the Invitrogen™ Attune™ NxT. The data were analyzed using FlowJo.

### Cell apoptosis assay

To determine cell apoptosis for both stable overexpression and knockdown cells, the Annexin V-AF647/DAPI Apoptosis Kit (#E-CK-A254, Elabscience) was employed. The harvested cells were incubated with Annexin V-AF647 along with DAPI Reagent in a binding buffer for 15 min at room temperature in the dark. Furthermore, to analyze apoptosis in cells exposed to SQLE inhibitors or squalene, the FITC Annexin V Apoptosis Detection Kit (#556547, BD Biosciences) was used as per the manufacturer’s instructions. Briefly, cells were incubated with FITC Annexin V and PI solution in a binding buffer at room temperature for 15 minutes in the dark. An Invitrogen™ Attune™ NxT flow cytometer was used to conduct flow cytometry, and the data were analyzed using FlowJo.

### RNA interference of gene expression

Small interfering RNA (siRNA) targeting SQLE and FDFT1 was purchased from Tsingke (Beijing, China). Transient transfections were carried out using Lipofectamine 3000 Transfection Reagent (#L3000015, Invitrogen). siRNA target sequences used were: SQLE (siSQLE-1: CUGGAGAUAUCAAGGAACUTT; siSQLE-2: CCACUGACAAUUCUCAUCUTT) and FDFT1 (siFDFT1-1: GGCAGUGAAGAUUCGGAAATT; siFDFT1-2: GCUACAAGUAUCUCAAUCATT).

### Transient plasmid overexpression

Src (#GM-MC-133241) and Akt (#GM-MC-133266) transient overexpressing plasmids were purchased from Genomeditech (Shanghai, China). Plasmid transfections were carried out using Lipofectamine 3000 Transfection Reagent (#L3000015, Invitrogen).

### Measurement of cholesterol levels

Cells (1 × 10^6^) were harvested, and total/free cholesterol levels were determined using the Cholesterol/Cholesteryl Ester Quantification Assay kit (#ab65359, Abcam).

### Lipid droplet staining

Lipid droplet staining assay was performed by BODIPY 493/503 (#D3922, Invitrogen™). In brief, the PC cells were incubated with 2 μM BODIPY staining solution at 37 °C for 30 min. The cells were then fixed with 4% paraformaldehyde for 30 min and counterstained with DAPI for 10 min. Images were generated by a Nikon AX/AX R confocal microscope. The quantification of BODIPY staining was performed by ImageJ.

### Filipin III staining

In all, 2 × 10^4^ PC cells were plated in 8-well chambered cover glass (#C8-1.5H-N, Cellvis). The cells were then fixed with 4% paraformaldehyde followed by incubation with 0.05 mg/ml Filipin III (Sigma, #SAE0087) staining solution at room temperature for 1.5 h. Furthermore, the nuclei were stained with SYTOX Deep Red stain (#P36990, Invitrogen). Fluorescence images of Filipin III staining were taken via a Nikon AX/AX R confocal microscope. The Filipin III staining quantification was analyzed by ImageJ.

### Lipid raft labeling

For the lipid raft labeling assay, the Vybrant® Alexa Fluor® 488 Lipid Raft Labeling Kit (#V34403, Invitrogen™) was utilized based on the manufacturer’s instructions. In brief, PC cells were labeled with fluorescent cholera toxin subunit B (CT-B) conjugate at 4 °C for 10 minutes. The CT-B-labeled lipid rafts were then crosslinked using anti-CT-B antibody for 15 minutes at 4 °C. Afterward, the cells were fixed using 4% formaldehyde at 4 °C for 15 min followed by permeabilization with 0.1% Triton X-100 for 10 min. Lipid raft labeling of cells was visualized using a Nikon AX/AX R confocal microscope, and ImageJ was used to quantify the CT-B staining.

### Animal experiments

The PUMCH Animal Care and Use Committees approved all the animal studies included in this project. Female BALB/c nude mice that were 6 weeks old were used in the mouse xenograft models. To examine the roles of SQLE in tumor growth, 5 × 10^6^ shNC and shSQLE AsPC-1 cells, 3 × 10^6^ shNC and shSQLE T3M4 cells, 5 × 10^6^ vector and SQLE overexpression MIA PaCa-2 cells or 1 × 10^7^ vector and SQLE overexpression PANC-1 cells were resuspended in 100 μL of PBS and Matrigel mixture (1:1) and then subcutaneously injected into the right flank of nude mice (*n* = 6 for AsPC-1 group, *n* = 5 for other groups). For the terbinafine treatment experiments, 3 × 10^6^ T3M4 cells or 5 × 10^6^ AsPC-1 cells were injected subcutaneously into the right flank of nude mice (*n* = 5 each group). Mice were randomly divided into two groups and treated with vehicle (PBS, orally, daily) or terbinafine (80 mg/kg, orally, daily) after cell injection for one week. Tumor size was measured every 3 days, and tumor volume was calculated using the formula: volume = 0.5 × length × width^2^. When the study concluded, the mice were euthanized, and the tumors were collected for analysis.

### RNA-seq analysis

Total RNA of shNC or shSQLE AsPC-1 cells (*n* = 4 each group) was extracted using TRIzol reagent (#15596026, Life Technologies). RNA sequencing (RNA-seq) was conducted on the Illumina HiSeq 2500 platform. The HISAT2 algorithm was used to map the sequenced reads to the human genome GRCh38 reference genome. We performed differential expression analysis (|Fold Change| > 1.5, padj < 0.05) between the shNC and shSQLE groups using the DESeq2 R package. The heatmap was created using the pheatmap R package to show 70 genes with the most statistically significant differences. Lastly, pathway enrichment of differential expression genes was conducted with the clusterProfiler R package, using the Kyoto Encyclopedia of Genes and Genomes (KEGG) database.

### Untargeted metabolomics analysis

In all, 1 × 10^7^ shNC or shSQLE AsPC-1 cells (*n* = 6 each) were harvested for untargeted metabolomics analysis. To extract metabolites, each sample was vortexed with 50 μL of ice-cold 80% methanol, treated immediately with liquid nitrogen, and then subjected to centrifugation at 10,000×*g* and 4 °C for 5 min. After repeating the process three times, we transferred the supernatant to a new microcentrifuge tube for subsequent LC-MS/MS analysis. The Vanquish UHPLC system from Thermo Fisher combined with an Orbitrap Q Exactive HF-X mass spectrometer (Thermo Fisher) operating in data-dependent acquisition mode facilitated the analysis. Differential metabolite analysis between the shNC and shSQLE groups was conducted (Log2(Fold Change) ≥ 1, *p* < 0.05, and variable importance in projection (VIP) value ≥ 1 using the OPLS-DA model).

### Bioinformatic analysis

We downloaded RNA-seq data for The Cancer Genome Atlas (TCGA) and the Genotype-Tissue Expression (GTEx) datasets from the University of California Santa Cruz (UCSC) Xena website (https://xenabrowser.net/datapages/). Microarray datasets (GSE15471, GSE62165, GSE62452, and GSE71729) were obtained from the Gene Expression Omnibus (GEO; http://www.ncbi.nlm.nih.gov/geo/). The MSigDB v7.4 (https://www.gsea-msigdb.org/gsea/msigdb) provided the gene set GOBP_REGULATION_OF_CHOLESTEROL_METABOLIC_PROCESS (detailed in Supplementary Table S2). Visualizing SQLE expression data and PC survival curves in TCGA combined GTEx were performed with Gene Expression Profiling Interactive Analysis 2 (GEPIA 2; http://gepia2.cancer-pku.cn/). The significantly enriched pathways for the differentially expressed genes between SQLE high and low expression groups of PC samples from the GSE62452 dataset were identified using Gene set enrichment analysis (GSEA). We obtained PC protein expression profiles from the Clinical Proteomic Tumor Analysis Consortium (CPTAC; https://cptac-data-portal.georgetown.edu). The R software (https://www.r-project.org) was used for statistical analysis and graphing.

### Statistical analysis

All data apart from involving animal were from at least three independent experiments and data were presented as mean ± standard error of the mean (SEM). Statistical analysis was performed by R software (version 4.0.3) and GraphPad Prism (version 8.0.1). Chi-square test was used to evaluate the correlations between SQLE expression levels and clinicopathological characteristics of the patients. Unpaired or paired two-tailed Student’s *t* tests, one-way analysis of variance (ANOVA), and two-way ANOVA were used to analyze the comparisons between groups. Survival analysis was conducted using the Kaplan–Meier method, and the significance of differences was calculated using the log-rank test. Pearson’s correlation was used to measure the correlations between genes. *P* < 0.05 was considered statistically significant.

## Results

### Identification of SQLE as a highly upregulated cholesterol metabolism gene in PC

**Fig. 1:**
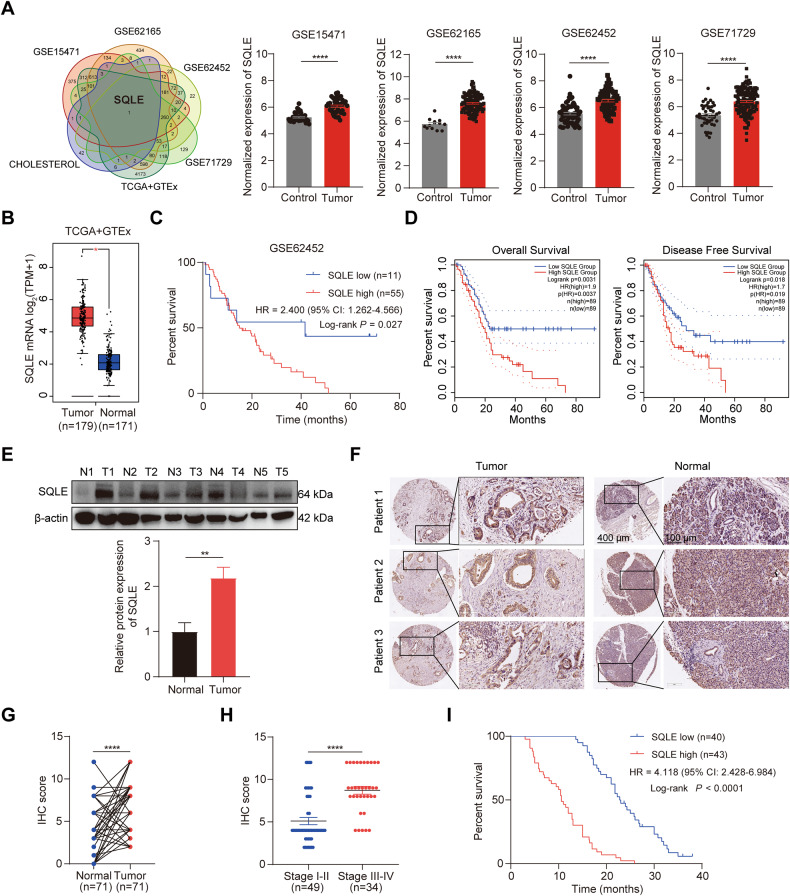
Identification of SQLE as a highly upregulated cholesterol metabolism gene in PC. **A**, **B** Identification of SQLE as the only upregulated gene in PC by integrating multiple Gene Expression Omnibus (GEO) datasets (GSE15471, GSE62165, GSE62452 and GSE71729) and The Cancer Genome Atlas (TCGA) and the Genotype-Tissue Expression (GTEx) using the gene set of regulation of cholesterol metabolic process from gene set enrichment analysis (GSEA) (False discovery rate (FDR) < 0.05 and |Fold Change| ≥ 1.5). **C** Kaplan–Meier survival analysis of overall survival based on the GSE62452 dataset in 11 PC patients with SQLE low expression and 55 PC patients with SQLE high expression. **D** Kaplan–Meier survival analysis of overall survival (left panel) and disease-free survival (right panel) of PC patients based on the TCGA dataset via the GEPIA 2 online database. **E** Western blotting images (top panel) and corresponding quantitative results (bottom panel) showing the expression of SQLE in 5 paired PC samples. **F** Representative images of immunohistochemical (IHC) staining of SQLE in tissue microarray (TMA) consisting of 71 paired PC samples. Scale bar: 400 μm (left images) and 100 μm (right images); **G** IHC scores of SQLE in TMA consisting of 71 paired PC samples. **H** IHC scores of SQLE in stage I-II (*n* = 49) and stage III-IV (*n* = 34) of PC samples in TMA. **I** Kaplan–Meier survival analysis of overall survival in TMA in 40 PC patients with SQLE low expression and 43 PC patients with SQLE high expression. *P < 0.05; **P < 0.01; ****P < 0.0001.

To identify essential regulators of cholesterol metabolism in PC, we analyzed several database subsets, including the GEO datasets GSE15471, GSE62165, GSE62452, GSE71729, and TCGA, as well as the GTEx to determine differentially expressed genes (False discovery rate (FDR) < 0.05 and |Fold Change| ≥ 1.5) using GSEA on the gene set of regulation of cholesterol metabolic process. We focused on upregulated genes that can be potential therapeutic targets. SQLE, which encodes the second-rate limiting enzyme in the cholesterol biosynthesis pathway, was the only differentially upregulated gene identified (Fig. 1A, B). Intriguingly, analyzing both GSE62452 and TCGA datasets revealed that PC patients with higher SQLE expression levels had shorter overall and disease-free survival rates (Fig. 1C, D). Additionally, our analyses indicated that compared to paired normal tissues, SQLE protein levels were significantly higher in PC tissues based on western blot analysis of five paired PC tissues and TMA analysis of 71 paired clinical PC tissue samples (Fig. 1E–G). Notably, in stages III–IV, SQLE expression was higher than in stages I–II (Fig. 1H), and a correlation exists between SQLE expression and cancer progression (Supplementary Table S3). Lastly, the overall survival of patients with high SQLE expression levels was shorter than those with low expression levels (Fig. 1I). Our findings indicate that upregulated SQLE expression is associated with a poor prognosis in PC.

### SQLE promotes PC cell proliferation in vitro

**Fig. 2:**
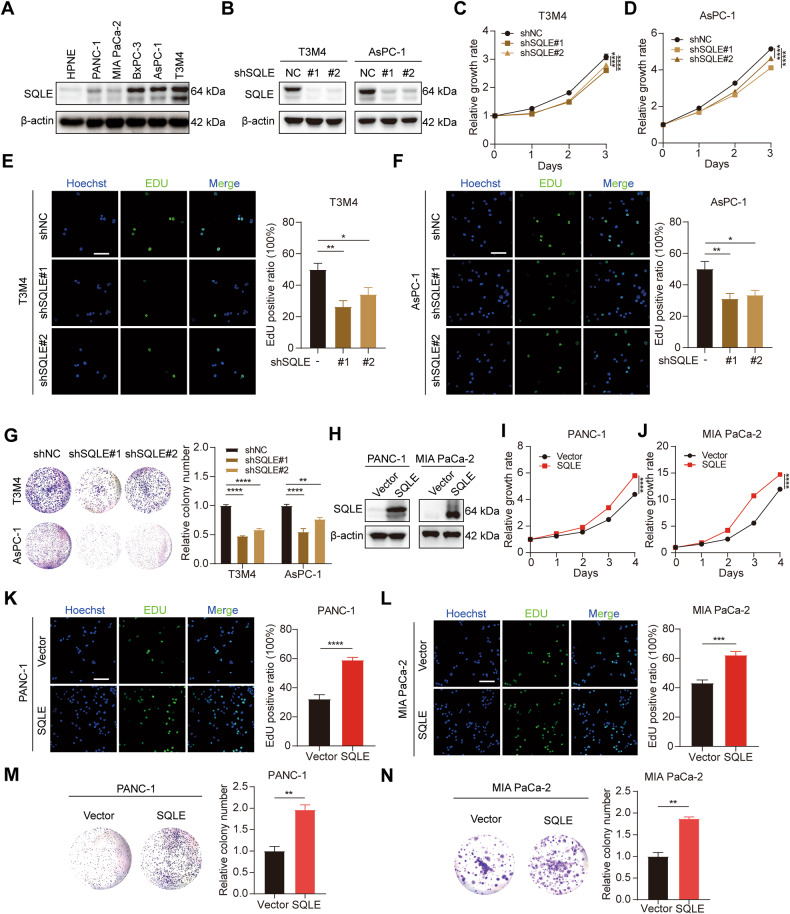
SQLE promotes PC cell proliferation in vitro. **A** Western blotting images showing SQLE protein levels in normal pancreatic ductal epithelial cell HPNE and PC cell lines including PANC-1, MIA PaCa-2, BxPC-3, AsPC-1, and T3M4. **B** Knockdown of SQLE protein levels of T3M4 and AsPC-1 cells were confirmed by Western blotting analysis. **C**, **D** Cell growth curves of T3M4 and AsPC-1 cells expressing control or SQLE shRNA. **E**, **F** Representative images of EdU staining (left panel) and EdU positive ratio (right panel) of T3M4 and AsPC-1 cells expressing control or SQLE shRNA. Scale bar: 100 μm. **G** Colony formation of T3M4 and AsPC-1 cells expressing control or SQLE shRNA. **H** Overexpression of SQLE protein levels of PANC-1 and MIA PaCa-2 cells were confirmed by Western blotting analysis. **I**, **J** Cell growth curves of PANC-1 and MIA PaCa-2 cells expressing vector or SQLE overexpression. **K**, **L** Representative images of EdU staining (left panel) and EdU positive ratio (right panel) of PANC-1 and MIA PaCa-2 cells expressing vector or SQLE overexpression. Scale bar: 100 μm. **M**, **N** Colony formation of PANC-1 and MIA PaCa-2 cells expressing vector or SQLE overexpression. *P < 0.05; **P < 0.01; ***P < 0.001; ****P < 0.0001.

To investigate the role of SQLE in PC, we examined the expression of SQLE in multiple cultured PC cell lines. All cell lines, including PANC-1, MIA PaCa-2, BxPC-3, AsPC-1, and T3M4, exhibited higher protein levels of SQLE than HPNE, a normal pancreatic ductal epithelial cell line (Fig. 2A). Accordingly, stable SQLE knockdown cell lines were constructed in T3M4 and AsPC-1 cells using shRNA interference guided by the expression levels of SQLE in PC cell lines (Fig. 2B and Supplementary Fig. S1A, B). This approach reduced the growth, EdU positive ratio, and colony formation of T3M4 and AsPC-1 cells (Fig. 2C–G). Subsequently, PANC-1 and MIA PaCa-2 cell lines overexpressing SQLE were constructed using a lentiviral transfection method. The efficiency of overexpression was confirmed by western blotting and real-time PCR (Fig. 2H and Supplementary Fig. S1C, D). The results showed that SQLE overexpression obviously increased the proliferation of PANC-1 and MIA PaCa-2 cells (Fig. 2I–N). Collectively, these data suggest that SQLE promotes the proliferation of PC cells in vitro.

### SQLE promotes PC cell proliferation in vivo

**Fig. 3:**
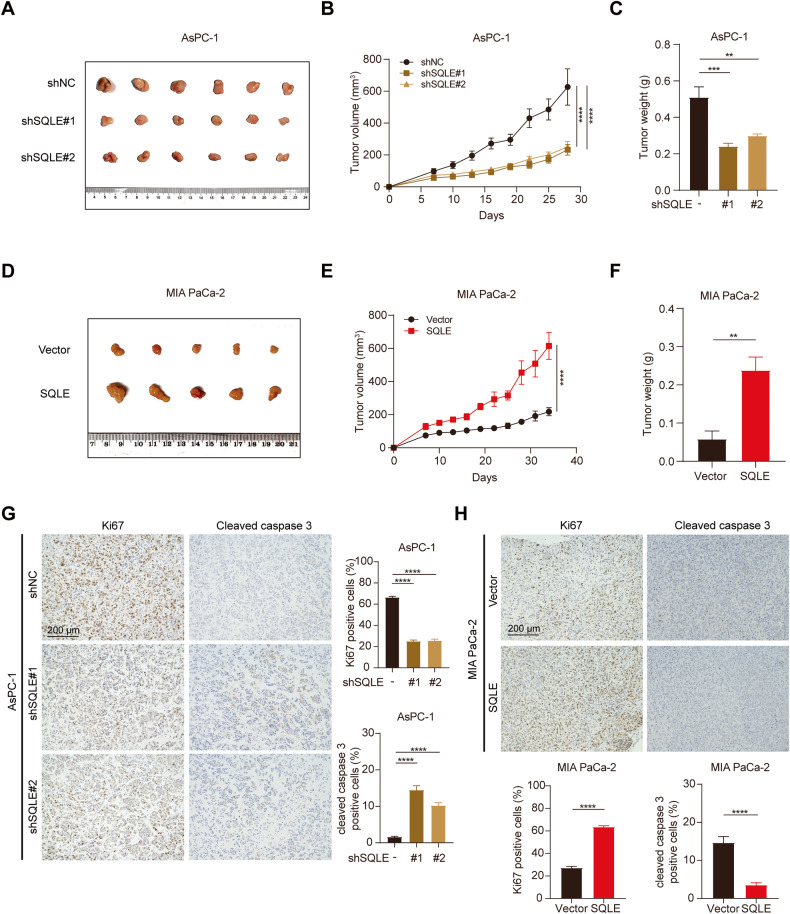
SQLE promotes PC cell proliferation in vivo. **A** Images of isolated tumors from the subcutaneous tumor nude mice model established using AsPC-1 cells expressing control or SQLE shRNA (*n* = 6 mice per group). **B**, **C** Tumor volume and weight in control and SQLE knockdown AsPC-1 groups. **D** Images of isolated tumors from the subcutaneous tumor nude mice model established using MIA PaCa-2 cells expressing vector or SQLE overexpression (*n* = 5 mice per group). **E**, **F** Tumor volume and weight in vector and SQLE overexpression MIA PaCa-2 groups. **G** Left, representative images of immunohistochemistry for Ki67 and cleaved caspase 3 staining in xenografts derived from AsPC-1 cells. Scale bar: 200 μm. Right, statistical analysis of Ki67 and cleaved caspase 3 positive cells. **H** Top, representative images of immunohistochemistry for Ki67 and cleaved caspase 3 staining in xenografts derived from MIA PaCa-2 cells. Scale bar: 200 μm. Bottom, statistical analysis of Ki67 and cleaved caspase 3 positive cells. **P < 0.01; ***P < 0.001; ****P < 0.0001.

To explore the role of SQLE in tumor growth in vivo, we established subcutaneous transplantation tumor models in nude mice using stable SQLE-knockdown AsPC-1 or T3M4 cells and stable SQLE-overexpressing MIA PaCa-2 or PANC-1 cells. Our findings demonstrated that SQLE knockdown significantly blunted the growth and weight of tumors derived from AsPC-1 or T3M4 cells (Fig. 3A–C and Supplementary Fig. S2A–C). In contrast, SQLE overexpression resulted in increased growth and weight of tumors derived from MIA PaCa-2 or PANC-1 cells (Fig. 3D–F and Supplementary Fig. S2D-F). Consistent with these findings, an immunohistochemical analysis for the proliferation (Ki67) and the apoptosis (cleaved caspase 3) indicated that inhibition of SQLE markedly decreased cell proliferation and augmented apoptosis in PC xenografts, whereas SQLE overexpression showed the opposite results (Fig. 3G, H and Supplementary Fig. S2G, H). Therefore, these results demonstrate that SQLE promotes pancreatic cancer tumor growth in vivo.

### SQLE promotes cell cycle progression and suppresses ER stress-triggered cell apoptosis

**Fig. 4:**
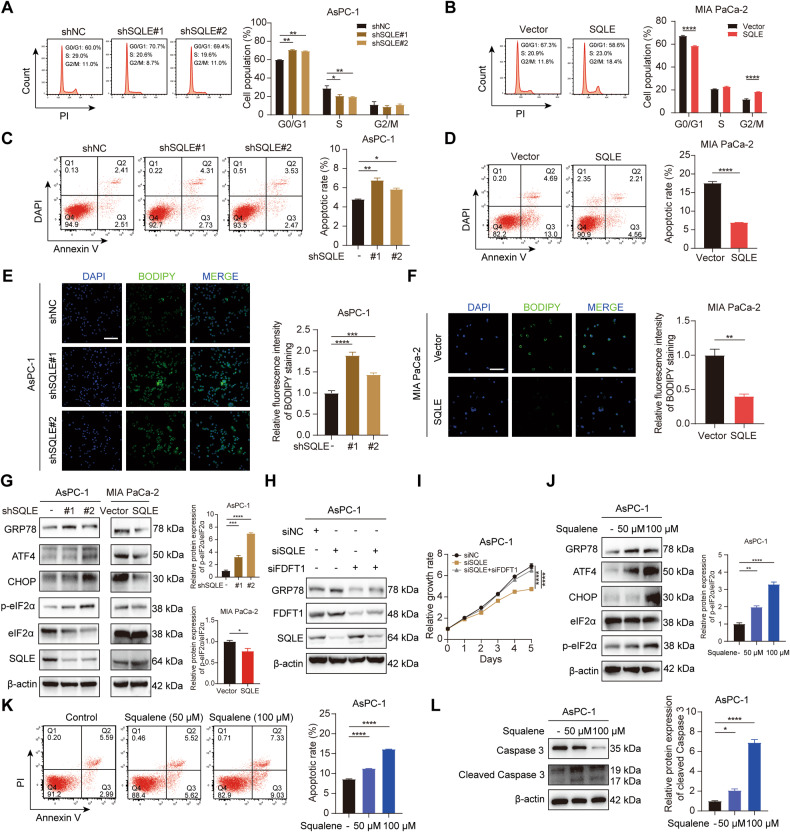
SQLE promotes cell cycle progression and suppresses ER stress-triggered cell apoptosis. **A** Cell cycle analysis of AsPC-1 cells expressing control or SQLE shRNA. **B** Cell cycle analysis of MIA PaCa-2 cells expressing vector or SQLE overexpression. **C** Cell apoptosis analysis of AsPC-1 cells expressing control or SQLE shRNA. **D** Cell apoptosis analysis of MIA PaCa-2 cells expressing vector or SQLE overexpression. **E**, **F** Detection of formation of lipid droplets using BODIPY 493/503 staining in AsPC-1 cells expressing control and SQLE shRNA or in MIA PaCa-2 cells expressing vector and SQLE overexpression. Left panel, representative images of BODIPY staining. Right panel, statistical analysis of relative fluorescence intensity of BODIPY staining. Scale bar: 100 μm. **G** Western blotting analysis showing proteins involved in the ER stress pathway after SQLE knockdown in AsPC-1 cells and overexpression in MIA PaCa-2 cells. **H** Western blotting images showing the protein levels of GRP78 in AsPC-1 cells with ±SQLE knockdown and ±FDFT1 knockdown. **I** Cell growth curves of AsPC-1 with ±SQLE knockdown and ±FDFT1 knockdown. **J** Western blotting analysis showing proteins involved in the ER stress pathway in AsPC-1 cells treated with 0, 50, and 100 μM squalene for 48 h. **K** Cell apoptosis analysis of AsPC-1 cells treated with 0, 50, and 100 μM squalene for 48 h. Left panel, representative images of flow cytometry. Right panel, statistical analysis of apoptotic rate of treated cells. **L** Western blotting analysis showing the expression of caspase 3 and cleaved caspase 3 in AsPC-1 cells treated with 0, 50, and 100 μM squalene for 48 h. *P < 0.05; **P < 0.01; ***P < 0.001; ****P < 0.0001.

We further investigated whether SQLE has a role in regulating cell cycle distribution and apoptosis by exploring the effects of SQLE knockdown or overexpression on AsPC-1, T3M4, MIA PaCa-2, and PANC-1 cells. Our results demonstrated that depletion of SQLE in AsPC-1 and T3M4 cells caused significant cell cycle arrest, an increase in the percentage of cells in the G0/G1 phase, and a decrease in the S or G2/M phase (Fig. 4A and Supplementary Fig. S3A). The overexpression of SQLE in MIA PaCa-2 and PANC-1 cells resulted in an increase in the percentage of cells in the S or G2/M phase, indicating cell cycle progression (Fig. 4B and Supplementary Fig. S3B). Furthermore, SQLE knockdown in AsPC-1 and T3M4 cells induced apoptosis (Fig. 4C and Supplementary Fig. S3C), while overexpression of SQLE in MIA PaCa-2 and PANC-1 cells suppressed apoptosis (Fig. 4D and Supplementary Fig. S3D). These results strongly indicate that SQLE plays a crucial role in regulating cell cycle distribution and apoptosis in PC cells.

To explore the potential mechanism behind the induction of cell apoptosis following SQLE inhibition, we focused on squalene, the substrate molecule of SQLE. Previous studies have reported that squalene accumulation following SQLE suppression can lead to the formation of lipid droplets budding from the ER membrane [22, 23]. We confirmed this observation by staining with BODIPY neutral lipid and observed a significant increase in lipid droplet formation due to SQLE knockdown but opposite in SQLE overexpression (Fig. 4E, F and Supplementary Fig. S3E, F). In addition, previous study has reported that lipid droplet formation is linked to the mitigation of ER stress caused by the accumulation of lipids or misfolded proteins [24]. Thus, we hypothesized that the appearance of increased lipid droplets was due to ER stress following the inhibition of SQLE and accumulation of squalene. To this end, we measured the changes in GRP78, an ER stress marker that is crucial in regulating the unfolded protein response (UPR). We found that the knockdown of SQLE significantly increased GRP78 expression and that this effect was impaired following SQLE overexpression (Fig. 4G). We further evaluated the activation of key downstream UPR transmembrane stress sensor PERK and subsequent phosphorylation of the eukaryotic translation initiation factor 2α (eIF2α) [25]. Our results showed that phosphorylated eIF2α-ATF4-activated CHOP transcription was increased in PC cells in which SQLE was knocked down, whereas SQLE overexpression impaired the activation of eIF2α-ATF4-CHOP axis (Fig. 4G). To confirm the effect of squalene accumulation following SQLE inhibition on ER stress, another cholesterol biosynthesis enzyme, farnesyl-diphosphate farnesyltransferase 1 (FDFT1), which produces squalene, was knocked down based on SQLE inhibition. Interestingly, the decreased expression of GRP78 was observed following FDFT1 suppression (Fig. 4H), underscoring our previous results. Consistently, knockdown of FDFT1 mitigated the inhibition of cell growth induced by SQLE knockdown (Fig. 4I and Supplementary Fig. S3G). In other experiments, the addition of exogenous squalene to the culture medium of PC cells activated the eIF2α-ATF4-CHOP axis through GRP78 regulation (Fig. 4J). To test the influence of ER stress due to squalene accumulation following SQLE inhibition on cell apoptosis, we carried out an apoptosis analysis by applying squalene. The apoptotic rate of AsPC-1 and T3M4 cells gradually increased with the rising dosage of squalene (Fig. 4K and Supplementary Fig. S3H). Concurrently, the final executor of apoptosis, cleaved caspase 3, was activated by squalene administration (Fig. 4L). Collectively, above results suggest that SQLE promotes cell cycle progression and the activation of GRP78-PERK-eIF2α-ATF4-CHOP-caspase 3 axis indicating ER stress-triggered cell apoptosis was induced by squalene accumulation following SQLE inhibition.

### SQLE promotes PC cell growth by activating downstream PI3K/Akt and MAPK signaling pathways

**Fig. 5:**
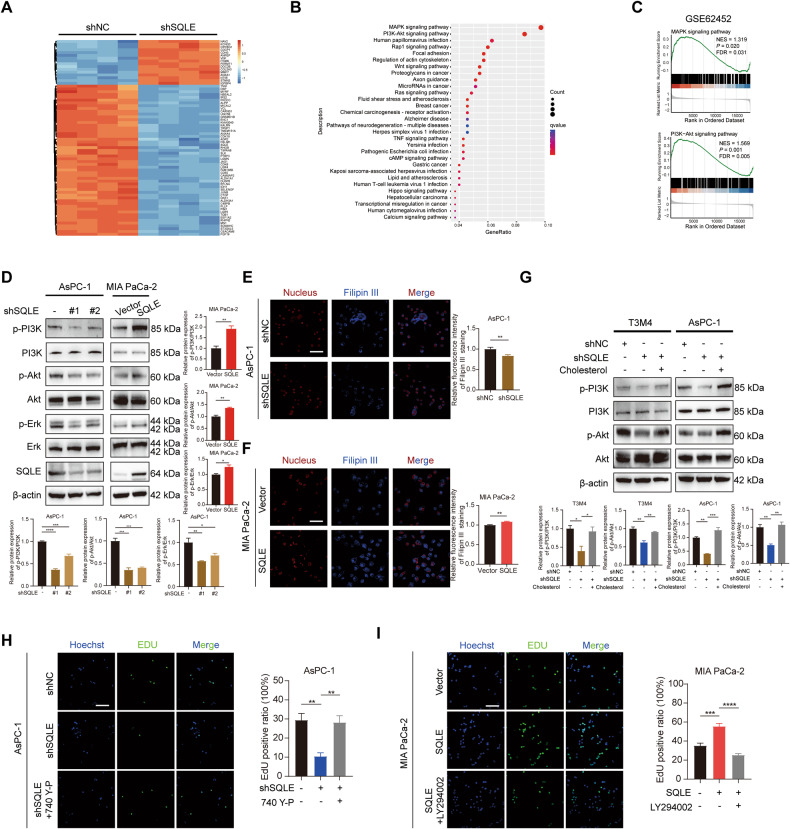
SQLE promotes PC cell growth by activating downstream PI3K/Akt and MAPK signaling pathways. **A** Heatmap showing 70 genes with the most statistically significant differences between SQLE knockdown AsPC-1 cells and control cells. **B** Kyoto Encyclopedia of Genes and Genomes (KEGG) analysis showing the statistically enriched pathways of differential expression genes between SQLE knockdown AsPC-1 cells and control cells. **C** Gene set enrichment analysis (GSEA) revealing significantly enriched both MAPK and PI3K/Akt signaling pathways in SQLE downstream target genes in the GSE62452 dataset. **D** Western blotting analysis showing the expression of PI3K, p-PI3K, Akt, p-Akt, Erk, and p-Erk after SQLE knockdown in AsPC-1 cells and overexpression in MIA PaCa-2 cells. **E** Filipin III staining showing the cellular free cholesterol content in AsPC-1 cells expressing control and SQLE shRNA. Left panel, representative images of Filipin III staining. Right panel, statistical analysis of relative fluorescence intensity of Filipin III staining. Scale bar: 100 μm. **F** Filipin III staining showing the cellular free cholesterol content in MIA PaCa-2 cells expressing vector or SQLE overexpression. Left panel, representative images of Filipin III staining. Right panel, statistical analysis of relative fluorescence intensity of Filipin III staining. Scale bar: 100 μm. **G** Western blotting analysis showing the protein levels of PI3K, p-PI3K, Akt, and p-Akt in T3M4 and AsPC-1 cells with ±SQLE knockdown and with/without 10 μM cholesterol supplementary. **H** Representative images of EdU staining (left panel) and EdU positive ratio (right panel) of AsPC-1 cells with ±SQLE knockdown and with/without 10 μM 740 Y-P treatment. Scale bar: 100 μm. **I** Representative images of EdU staining (left panel) and EdU positive ratio (right panel) of MIA PaCa-2 cells with ±SQLE overexpression and with/without 10 μM LY294002 treatment. Scale bar: 100 μm. *P < 0.05; **P < 0.01; ***P < 0.001; ****P < 0.0001.

To explore the mechanism by which SQLE promotes PC cell growth, we performed RNA-seq to analyze differential gene expression between SQLE gene knockdown AsPC-1 cells and the control cells (Fig. 5A). KEGG analysis of the data showed that the MAPK and PI3K/Akt signaling pathways were significantly enriched (Fig. 5B). Analyzing GSE62452 dataset using GSEA also revealed that both MAPK and PI3K/Akt signaling pathways were significantly enriched in SQLE downstream target genes (Fig. 5C). Western blotting analysis confirmed that knockdown of SQLE reduced the expression of phospho-PI3K, phospho-Akt, and phospho-Erk, while SQLE overexpression in PC cells increased their expression (Fig. 5D and Supplementary Fig. S4A). A growing body of evidence has demonstrated a close relationship between cellular cholesterol levels and PI3K/Akt pathway activation [26, 27]. As the rate-limiting enzyme of the cholesterol biosynthesis pathway, the influence of SQLE interference on the changes of cellular cholesterol levels was determined. We observed that the concentration of total and free cholesterol increased following SQLE overexpression and decreased upon SQLE knockdown (Supplementary Fig. S4B, C). Filipin III staining intensity also showed that cellular free cholesterol content was significantly decreased in SQLE knockdown AsPC-1 and T3M4 cells, while SQLE overexpression caused an increase in cellular free cholesterol content (Fig. 5E, F and Supplementary Fig. S4D, E). We, therefore, hypothesized that SQLE promotes the induction of the PI3K/Akt pathway by influencing cellular cholesterol levels. The results showed that cholesterol repletion partially rescued the expression of phosphorylation of PI3K and Akt induced by SQLE knockdown (Fig. 5G). Additionally, we treated transfected cells with PI3K/Akt signaling agonist 740 Y-P or antagonist LY294002 to examine the role of PI3K/Akt signaling on cell growth. We found that 740 Y-P partially rescued the growth of AsPC-1 and T3M4 cells induced by SQLE knockdown, while LY294002 inhibited the increased growth of SQLE overexpression MIA PaCa-2 and PANC-1 cells using EdU and colony formation assays (Fig. 5H, I and Supplementary Fig. S4F, I). In conclusion, these results indicate that PI3K/Akt signaling is critical for promoting SQLE-mediated growth of PC cells.

### SQLE regulates the Src/PI3K/Akt cascade via cholesterol-dependent lipid rafts

**Fig. 6:**
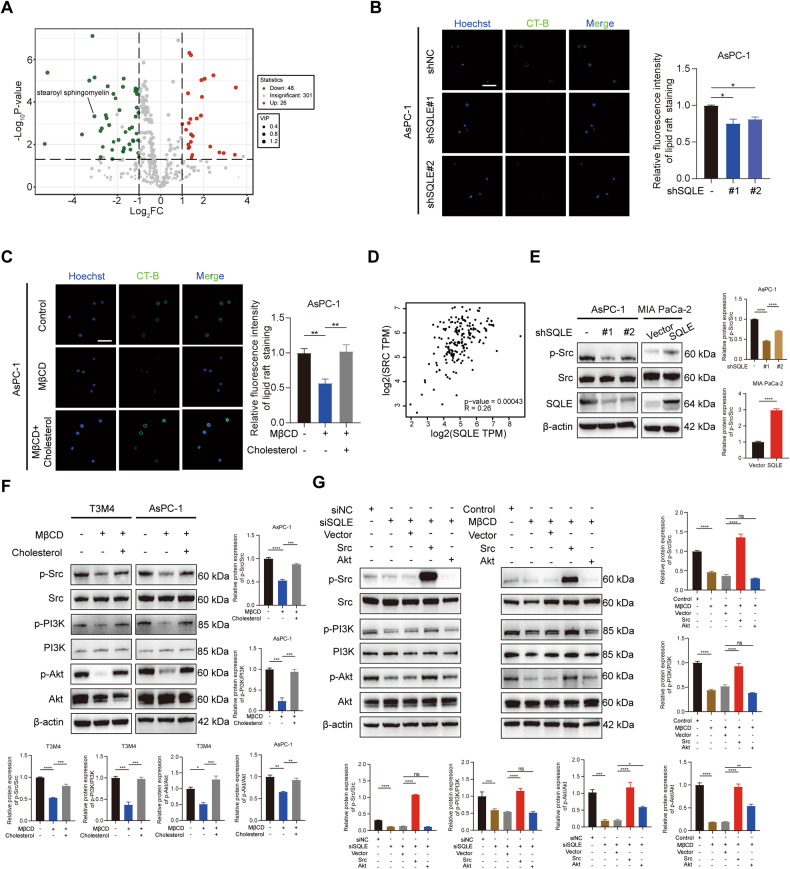
SQLE regulates the Src/PI3K/Akt cascade via cholesterol-dependent lipid rafts. **A** Volcano plots showing differential metabolites between SQLE knockdown AsPC-1 cells and control cells. Stearoyl sphingomyelin exhibited a significant decline (Log_2_(Fold Change) = −3.06, *P* < 0.001, and VIP value = 1.50) in SQLE knockdown cells. **B** CT-B staining showing the contents of lipid rafts in AsPC-1 cells expressing control or SQLE shRNA. Left panel, representative images of CT-B staining. Right panel, statistical analysis of relative fluorescence intensity of CT-B staining. Scale bar: 100 μm. **C** CT-B staining showing the contents of lipid rafts in AsPC-1 cells after treatment with 10 mM MβCD for 30 min or 10 mM MβCD for 30 min + 10 μM cholesterol overnight. Left panel, representative images of CT-B staining. Right panel, statistical analysis of relative fluorescence intensity of CT-B staining. Scale bar: 100 μm. **D** Pearson’s correlation analysis showing correlation between SQLE and SRC via the GEPIA 2 online database. **E** Western blotting analysis showing the protein levels of Src and p-Src after SQLE knockdown in AsPC-1 cells and overexpression in MIA PaCa-2 cells. **F** Western blotting analysis showing the expression of Src, p-Src, PI3K, p-PI3K, Akt, and p-Akt in T3M4 and AsPC-1 cells after treatment with 10 mM MβCD for 30 min or 10 mM MβCD for 30 min + 10 μM cholesterol overnight. **G** Western blotting analysis showing the expression of Src, p-Src, PI3K, p-PI3K, Akt, and p-Akt in T3M4 cells with ±SQLE knockdown (left panel) or treatment with 10 mM MβCD for 30 min (right panel) and ±Src overexpression or ±Akt overexpression. *P < 0.05; **P < 0.01; ***P < 0.001; ****P < 0.0001.

We further conducted a metabolomics analysis to determine the mediator between SQLE and PI3K/Akt signaling, which revealed the downregulation of 48 metabolites and the upregulation of 26 metabolites upon SQLE knockdown (Fig. 6A). Interestingly, we observed a significant downregulation of stearoyl sphingomyelin in SQLE knockdown cells (Fig. 6A). Previous studies have suggested that both sphingomyelin and cholesterol are essential lipids for the formation of specialized membrane domains called lipid rafts [28]. As we have observed that cholesterol levels could be influenced by SQLE, we speculated that SQLE dysregulation might impact lipid rafts’ function. As anticipated, knockdown of SQLE decreased lipid rafts staining intensity through a fluorescently labeled CT-B, while overexpression of SQLE significantly increased lipid rafts staining intensity (Fig. 6B and Supplementary Fig. S5A–C). We further observed that treatment of PC cells with methyl-β-cyclodextrin (MβCD), which specifically destructs lipid rafts, significantly depleted lipid rafts staining intensity, while cholesterol supplementation restored its function (Fig. 6C and Supplementary Fig. S5D).

It has been shown that lipid rafts play important roles in cellular signal transduction and Src family kinases (SFKs) are strongly associated with lipid rafts [29, 30]. We noted that the expression of Src was increased in the SQLE high expression group by analyzing the CPTAC dataset (Supplementary Fig. S5E), and SQLE and SRC expressions in PC tissues showed a positive correlation (Fig. 6D). We assumed that Src was the signal transducer responsible for mediating SQLE-induced-lipid rafts-regulated downstream signal transduction. As expected, Western blot confirmed that SQLE knockdown decreased the expression of phospho-Src in AsPC-1 cells, while SQLE overexpression in MIA PaCa-2 cells increased its expression (Fig. 6E). In addition, MβCD treatment decreased the expression of phospho-Src, phospho-PI3K, and phospho-Akt, while replenishment of cholesterol restored them compared to control cells (Fig. 6F). These findings suggest that lipid rafts’ deregulation can influence the activation of Src and PI3K/Akt pathway. To further confirm whether Src contributes to lipid rafts-induced PI3K/Akt activation, we overexpressed Src or Akt in PC cells with SQLE interference or MβCD treatment. The results showed that overexpression of Src restored the decreased expression of phospho-PI3K and phospho-Akt induced by SQLE interference or MβCD treatment, whereas overexpression of Akt failed to restore the decreased expression of phospho-Src, indicating Src was the inducer of PI3K/Akt signaling pathway (Fig. 6G). Thus, our results demonstrate that SQLE regulates the Src/PI3K/Akt cascade via cholesterol-dependent lipid rafts.

### SQLE inhibitors terbinafine and NB-598 suppress proliferation, induce cell cycle arrest, and activate ER stress-triggered apoptosis of PC cells in vitro

**Fig. 7:**
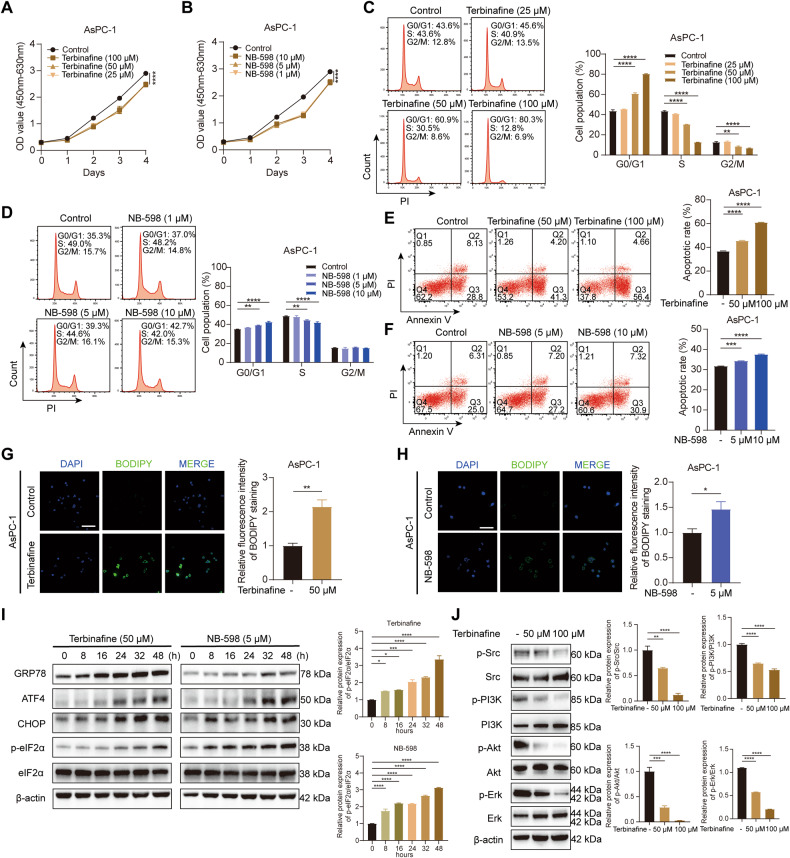
SQLE inhibitors terbinafine and NB-598 suppress proliferation, induce cell cycle arrest and activate ER stress-triggered apoptosis of PC cells in vitro. **A** Cell growth curves of AsPC-1 cells treated with 0, 25, 50, and 100 μM terbinafine for 4d. **B** Cell growth curves of AsPC-1 cells treated with 0, 1, 5, and 10 μM NB-598 for 4d. **C** Cell cycle analysis of AsPC-1 cells treated with 0, 25, 50, and 100 μM terbinafine for 48 h. **D** Cell cycle analysis of AsPC-1 cells treated with 0, 1, 5, and 10 μM NB-598 for 48 h. **E** Cell apoptosis analysis of AsPC-1 cells treated with 0, 50, and 100 μM terbinafine for 48 h. **F** Cell apoptosis analysis of AsPC-1 cells treated with 0, 5, and 10 μM NB-598 for 48 h. **G** Detection of formation of lipid droplets using BODIPY 493/503 staining in AsPC-1 cells treated with 0 and 50 μM terbinafine for 48 h. Left panel, representative images of BODIPY staining. Right panel, statistical analysis of relative fluorescence intensity of BODIPY staining. Scale bar: 100 μm. **H** Detection of formation of lipid droplets using BODIPY 493/503 staining in AsPC-1 cells treated with 0 and 5 μM NB-598 for 48 h. Left panel, representative images of BODIPY staining. Right panel, statistical analysis of relative fluorescence intensity of BODIPY staining. Scale bar: 100 μm. **I** Western blotting analysis showing proteins involved in the ER stress pathway in AsPC-1 cells treated with 50 μM terbinafine (left panel) and 5 μM NB-598 (right panel) for 0, 8, 16, 24, 32, and 48 h. **J** Western blotting analysis showing the expression of Src, p-Src, PI3K, p-PI3K, Akt, p-Akt, Erk, and p-Erk in AsPC-1 cells treated with 0, 50, and 100 μM terbinafine for 48 h. *P < 0.05; **P < 0.01; ***P < 0.001; ****P < 0.0001.

As SQLE has been shown to promote cell proliferation and tumor growth in PC, we assessed the potential clinical application of SQLE inhibitors. PC cells were treated with terbinafine, which is widely used to treat fungal infections, or NB-598, a highly specific SQLE inhibitor for mammals. Treatment with either of the compounds significantly blunted the growth of AsPC-1 and T3M4 cells in vitro in a dose-dependent manner (Fig. 7A, B and Supplementary Fig. S6A, B). Terbinafine or NB-598 treatment also induced G0/G1 phase arrest with concurrent decreases in S phase and G2/M phase compared to the control group (Fig. 7C, D and Supplementary Fig. S6C, D). Consistent with the observed findings, NB-598 treatment suppressed the CDK4 protein expression but increased the expression of cell cycle inhibitors p21 and p27 (Supplementary Fig. S6E). Likewise, SQLE inhibitors dose-dependently induced apoptosis of AsPC-1 and T3M4 cells (Fig. 7E, F and Supplementary Fig. S6F, G).

Since SQLE knockdown has been validated to activate the formation of lipid droplets and ER stress caused by squalene accumulation, we further examined how SQLE inhibitors affected these effects. As anticipated, terbinafine or NB-598 treatment increased the intensity of BODIPY staining in AsPC-1 and T3M4 cells, indicating increased lipid droplet formation (Fig. 7G, H and Supplementary Fig. S6H, I). In addition, terbinafine or NB-598 treatment increased the expression levels of GRP78, phosphorylated eIF2α, ATF4, and CHOP in a time-dependent manner (Fig. 7I). Interestingly, the protein levels of SQLE were continuously increased by terbinafine or NB-598 treatment, which strengthened previous findings that accumulation of squalene stabilizes the N-terminal region of SQLE, attenuating ubiquitination-mediated degradation (Supplementary Fig. S6J). The results described above suggested that apoptosis mediated by ER stress could be induced by squalene accumulation following SQLE inhibitors. Finally, we examined the changes of MAPK signaling pathway and Src/PI3K/Akt cascade following SQLE inhibitor treatment. Treatment with terbinafine induced a dose-dependent decrease in the expression of phospho-Erk, phospho-Src, phospho-PI3K, and phospho-Akt, which suggests that SQLE regulates the MAPK signaling pathway and Src/PI3K/Akt cascade (Fig. 7J).

### SQLE inhibitor attenuates PC tumor growth

**Fig. 8:**
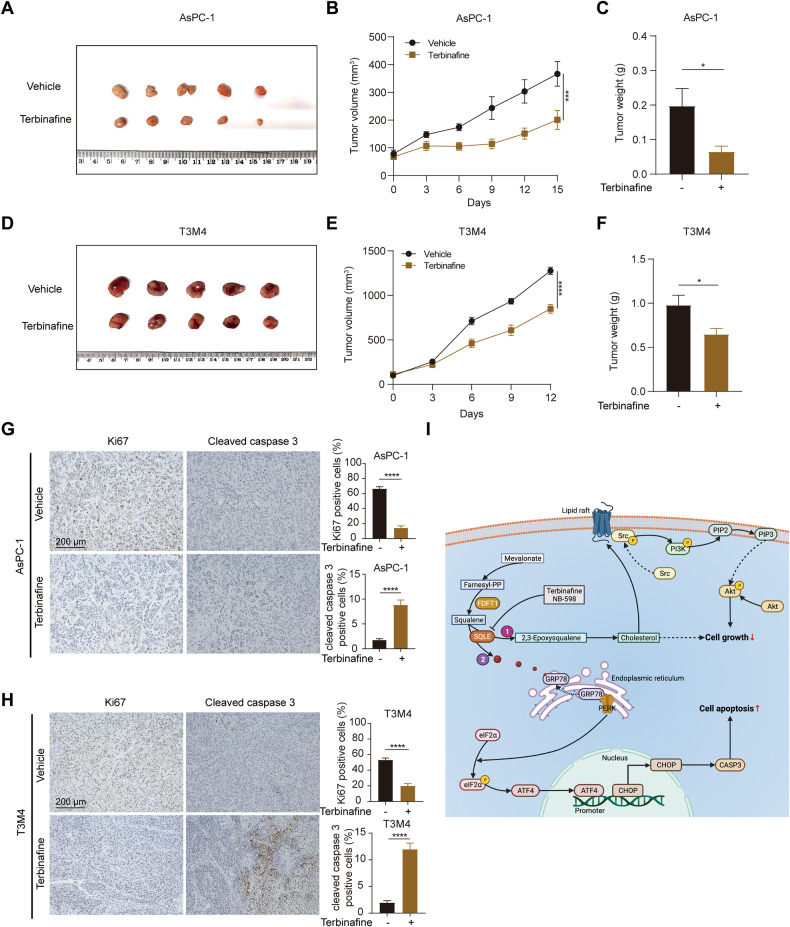
SQLE inhibitor attenuates PC tumor growth. **A** Images of isolated tumors from the subcutaneous tumor nude mice model established using AsPC-1 cells treated with vehicle (PBS, orally, daily) or terbinafine (80 mg/kg, orally, daily) (*n* = 5 mice per group). **B**, **C** Tumor volume and weight in vehicle or terbinafine-treated AsPC-1 groups. **D** Images of isolated tumors from the subcutaneous tumor nude mice model established using T3M4 cells treated with vehicle (PBS, orally, daily) or terbinafine (80 mg/kg, orally, daily) (*n* = 5 mice per group). **E**, **F** Tumor volume and weight in vehicle or terbinafine-treated T3M4 groups. **G** Left, representative images of immunohistochemistry for Ki67 and cleaved caspase 3 staining in xenografts derived from AsPC-1 cells. Scale bar: 200 μm. Right, statistical analysis of Ki67 and cleaved caspase 3 positive cells. **H** Left, representative images of immunohistochemistry for Ki67 and cleaved caspase 3 staining in xenografts derived from T3M4 cells. Scale bar: 200 μm. Right, statistical analysis of Ki67 and cleaved caspase 3 positive cells. **I** A proposed working model. Inhibition of SQLE attenuates cell growth and induces cell apoptosis in PC in two ways. On the one hand, SQLE inhibition decreases cellular cholesterol content and destructs the function of lipid rafts. Inactivated lipid rafts could not serve as a signal transduction platform to stimulate Src/PI3K/Akt cascade, attenuating cell growth. On the other hand, suppression of SQLE causes substrate squalene accumulation in the endoplasmic reticulum (ER). Squalene could further interact with ER stress regulator GRP78, inducing PERK-eIF2α-ATF4-CHOP-caspase 3 axis-mediated cell apoptosis. Inhibition of SQLE by terbinafine or NB-598 might serve as a promising treatment strategy for PC. This figure was created on BioRender.com with permission for publication. *P < 0.05; ***P < 0.001; ****P < 0.0001.

We established mouse xenograft models bearing AsPC-1 and T3M4 cells to further evaluate the anti-tumor efficacy of terbinafine. The terbinafine administration significantly suppressed tumor growth and weight in a manner consistent with in vitro findings (Fig. 8A–F). Notably, we observed no significant differences in the body weight of mice treated with terbinafine or vehicle (Supplementary Fig. S7A, B). We conducted IHC analysis for Ki67 and cleaved caspase 3, revealing that terbinafine administration markedly reduced cell proliferation and increased cell apoptosis of PC xenografts (Fig. 8G, H). Our findings suggest that pharmacological inhibition of SQLE is a promising therapeutic approach for PC.

## Discussion

Malignant cancer cells often exhibit altered metabolic patterns to thrive in the harsh tumor microenvironment (TME), providing potential therapeutic vulnerabilities. Accumulating evidence suggests that interference of deregulated cancer metabolism by targeting metabolic enzymes and their associated metabolites disrupts tumor initiation and progression [31]. In current study, we identified SQLE as a master cholesterol metabolism regulator in PC growth. We found that SQLE was elevated in PC tissues compared to normal tissues, and patients with high expression of SQLE have a shorter overall survival. Further functional experiments revealed that SQLE promoted cell proliferation, fostered cell cycle progression, and inhibited cell apoptosis in vitro, while enhancing tumor growth in vivo. Mechanistically, on the one hand, suppression of SQLE attenuates cell growth by regulating the Src/PI3K/Akt signaling via cholesterol-dependent lipid rafts. On the other hand, squalene accumulation following SQLE inhibition regulates the activation of ER stress and subsequent induction of cell apoptosis. Moreover, SQLE inhibitors could serve potential therapeutical approach with the role in suppressing PC growth (Fig. 8I).

In recent years, multiple studies have demonstrated that altered cholesterol metabolism contributes to the regulation of various biological processes of tumors. It has also been revealed that manipulating cholesterol metabolism can inhibit tumor growth as well. Most mammalian cells maintain cholesterol homeostasis by dynamically balancing cholesterol biosynthesis, uptake, export, and esterification [9]. Hence, dysregulation of these pathways has been shown to play a crucial role in promoting the development and progression of PC. For instance, PC cell proliferation is decreased by blocking the low-density lipoprotein receptor (LDLR), which is responsible for the uptake of low-density lipoprotein (LDL) from the circulation [32]. It was recently demonstrated that plasma samples of PC patients exhibited a significantly decreased cholesterol efflux capacity compared with healthy controls and high-density lipoprotein (HDL)-mediated cholesterol efflux results in reduction of PC cell growth and induction of cell apoptosis [33]. Pharmacological or genetic inhibition of cholesterol esterification markedly inhibited the growth and metastasis of PC [34]. In terms of cholesterol biosynthesis, the gene expression of mevalonate pathway which is a key component of cholesterol biosynthetic pathway was elevated in KRAS mutant acinar cells, supporting the formation of acinar-to-ductal metaplasia (ADM) [35]. SQLE, the second rate-limiting enzyme in the cholesterol biosynthesis, was found to be elevated in several cancers such as hepatocellular carcinoma, breast cancer, head and neck squamous cell carcinoma, and prostate cancer, contributing to tumor progression and poorer outcomes for patients [17–20]. In this study, we showed that SQLE was upregulated in PC, and patients with high SQLE expression levels had shorter survival rates. Interestingly, there are discrepancies regarding the role of SQLE in colorectal cancer (CRC). Two studies have reported that overexpression of SQLE promotes CRC cell proliferation [36, 37], while Jun et al.’s findings suggest that reduced SQLE, fueled by cholesterol accumulation, aggravates CRC progression and metastasis [38]. Notably, SQLE was upregulated in CRC when compared to normal tissues, but significantly decreased during CRC progression [38]. This implies that SQLE has a dual role in different stages of cancer, particularly in CRC, where it has a tumor-promoting function in tumorigenesis but a tumor-suppressing effect in progression.

We investigated the mechanism underlying the growth-promoting effect of SQLE in PC and discovered that SQLE promotes both cell proliferation and inhibits apoptosis of PC cells. We focused specifically on the accumulation of SQLE substrate squalene after SQLE inhibition, which has been hypothesized to promote lipid droplet budding from the ER membrane by localizing in the center of the lipid bilayer [22, 23, 39]. In our study, SQLE inhibition significantly increased lipid droplet formation. Current research reveals that maintaining a balance between protein and lipid synthesis is vital for the functioning of the ER, and, consequently, both misfolded protein and lipid accumulation are responsible for ER stress. Typically, when ER stress is induced, lipid droplets play a protective role in toxic lipid storage, thus their accumulation increases [40]. Our study found an activation of GRP78-PERK-eIF2α-ATF4-CHOP axis in a characterized ER stress pathway, as well as induction of PC cell death with the formation of lipid droplets after SQLE inhibition. Our results are consistent with a recent study on breast cancer and non-small cell lung cancer [41]. Nonetheless, the role of squalene may be context-dependent as demonstrated by Garcia-Bermudez et al., who reported that squalene protects ALK^+^ anaplastic large-cell lymphoma cells from lipid peroxidation-induced ferroptosis [42]. Additionally, accumulation of cholesterol in CRC cells, regardless of squalene, contributes to their survival [38].

We conducted RNA sequencing analysis between SQLE knockdown and SQLE negative control cells to investigate the downstream signaling pathway influenced by SQLE in PC. With our KEGG analysis and GSEA of a GEO dataset, we validated that both the MAPK and PI3K/Akt signaling pathways were significantly enriched. These findings were further validated by Western blotting analysis. Additionally, we established a close correlation between changes in cellular cholesterol content and SQLE expression levels. Our functional experiments validated that the oncogenic roles of SQLE in PC were facilitated by downstream PI3K/Akt signaling, which is consistent with findings in head and neck squamous cell carcinoma [19]. Further untargeted metabolomics analysis revealed that stearoyl sphingomyelin expression was significantly reduced upon SQLE inhibition. Lipid rafts, consisting of sphingomyelin and cholesterol, are critical for signal transduction. Intriguingly, a recent study suggested that in clear cell renal cell carcinoma, cholesterol import mediated by Scavenger Receptor B1 (SCARB1) is imperative for lipid raft homeostasis and the PI3K/Akt pathway activation [27]. Our study demonstrated significant changes in the function of lipid rafts regulated by SQLE. Src, a member of nonreceptor tyrosine kinases SFKs, plays a crucial role in malignant progression for various cancers, as overexpression/activation promotes malignancy [43]. Interestingly, the function of Src can be spatially regulated through a shift between the cytosolic space and intracellular membrane, active Src being translocated into lipid rafts via scaffold proteins to activate a specific downstream signaling pathway [44, 45]. Furthermore, our study found a positive correlation between SQLE and SRC expression levels based on publicly available PC datasets. Additionally, we conducted a series of rescue experiments that confirmed that Src acted as the upstream inducer of the PI3K/Akt signaling pathway, depending on the function of lipid rafts.

To evaluate the effectiveness of SQLE inhibitors in treating PC, we investigated the efficacy of terbinafine, a commonly used antifungal medication known to exert growth-inhibitory effects on multiple cancers [17–19]. In this study, we demonstrated that terbinafine treatment inhibited cell proliferation, induced cell cycle arrest and apoptosis in vitro, delayed tumor growth in vivo, and suppressed the MAPK and Src/PI3K/Akt signaling pathways. Notably, it was also found that a specific mammalian SQLE inhibitor NB-598 had a more potent inhibitory effect on PC growth than terbinafine at a lower dosage. These results indicate that SQLE inhibitors have potential as an effective treatment strategy for PC. Moreover, a synergistic anti-tumor effect of combining terbinafine with chemotherapeutic agents or targeted therapies has been shown in various cancer types [37, 46]. A recent study demonstrated that terbinafine enhanced chemotherapeutic sensitivity in PC cells, which needs to be validated in vivo in the future [47]. However, due to the high metabolic plasticity of PC, cells may utilize compensatory mechanisms, such as other nutrients or metabolic pathways, to sustain growth in the unique TME. Consistent with this idea, we found that replenishing exogenous cholesterol rescued the anti-tumor effect of SQLE interference. Furthermore, we observed that sterol regulatory element-binding protein 2 (SREBP2) and downstream LDLR expression levels could be partially induced following SQLE inhibition (data not shown), which may highlight compensatory mechanisms. Therefore, combining SQLE inhibitors and cholesterol uptake suppression such as Liver X Receptor (LXR) agonists which could cause LDLR degradation and cholesterol efflux may have synergistic therapeutic efficacy, which warrants further study.

Meanwhile, this study’s limitations need to be considered critically. First, the underlying molecular mechanism by which squalene induces ER stress and whether other UPR pathways are involved remains elusive. Secondly, the present study mainly focuses on the role of SQLE in tumorigenesis and cell growth. However, a positive correlation between SQLE expression levels, lymph node invasion, and distant metastasis in the TMA analysis was also observed. Whether SQLE plays a role in PC progression is yet to be investigated extensively. Additionally, we utilized human PC cell line-derived xenografts for our in vivo study. However, more preclinical models, such as PC patient-derived xenograft (PDX) models and transgenic mice, are required to unveil the roles of SQLE in PC.

## Conclusions

In conclusion, our study demonstrated that SQLE as a key cholesterol metabolism regulator that promoted cell growth and tumorigenesis of PC. The oncogenic roles of SQLE are achieved by promoting lipid rafts-regulated Src/PI3K/Akt signaling pathways and weakened squalene accumulation-induced ER stress. Encouragingly, using SQLE inhibitors in both in vitro and in vivo models significantly inhibited PC growth, suggesting SQLE as a promising therapeutic target for PC.

## Supplementary information

Supplementary Figure S1

Supplementary Figure S2

Supplementary Figure S3

Supplementary Figure S4

Supplementary Figure S5

Supplementary Figure S6

Supplementary Figure S7

Supplementary Figure legends

Supplementary Table S1

Supplementary Table S2

Supplementary Table S3

Original western blots

Reproducibility Checklist

## Data Availability

The data that support the findings of this study are available from the corresponding author upon reasonable request.
