# Supplementary figures and images for: SQLE promotes pancreatic cancer growth by attenuating ER stress and activating lipid rafts-regulated Src/PI3K/Akt signaling pathway
Source: Cell Death Dis. 2023 Aug 4;14(8):497. doi: 10.1038/s41419-023-05987-7 (PMC10403582; doi:10.1038/s41419-023-05987-7)

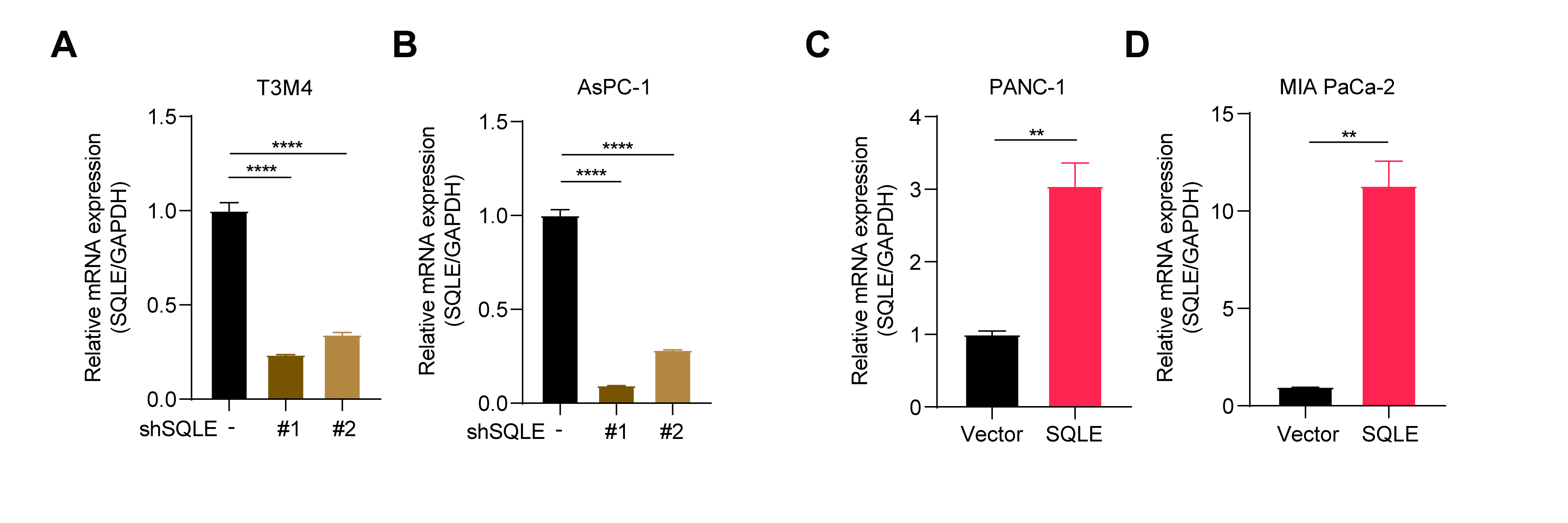

Supplement: Supplementary file 1 — Supplementary Figure S1 [file 41419_2023_5987_MOESM1_ESM.tif]

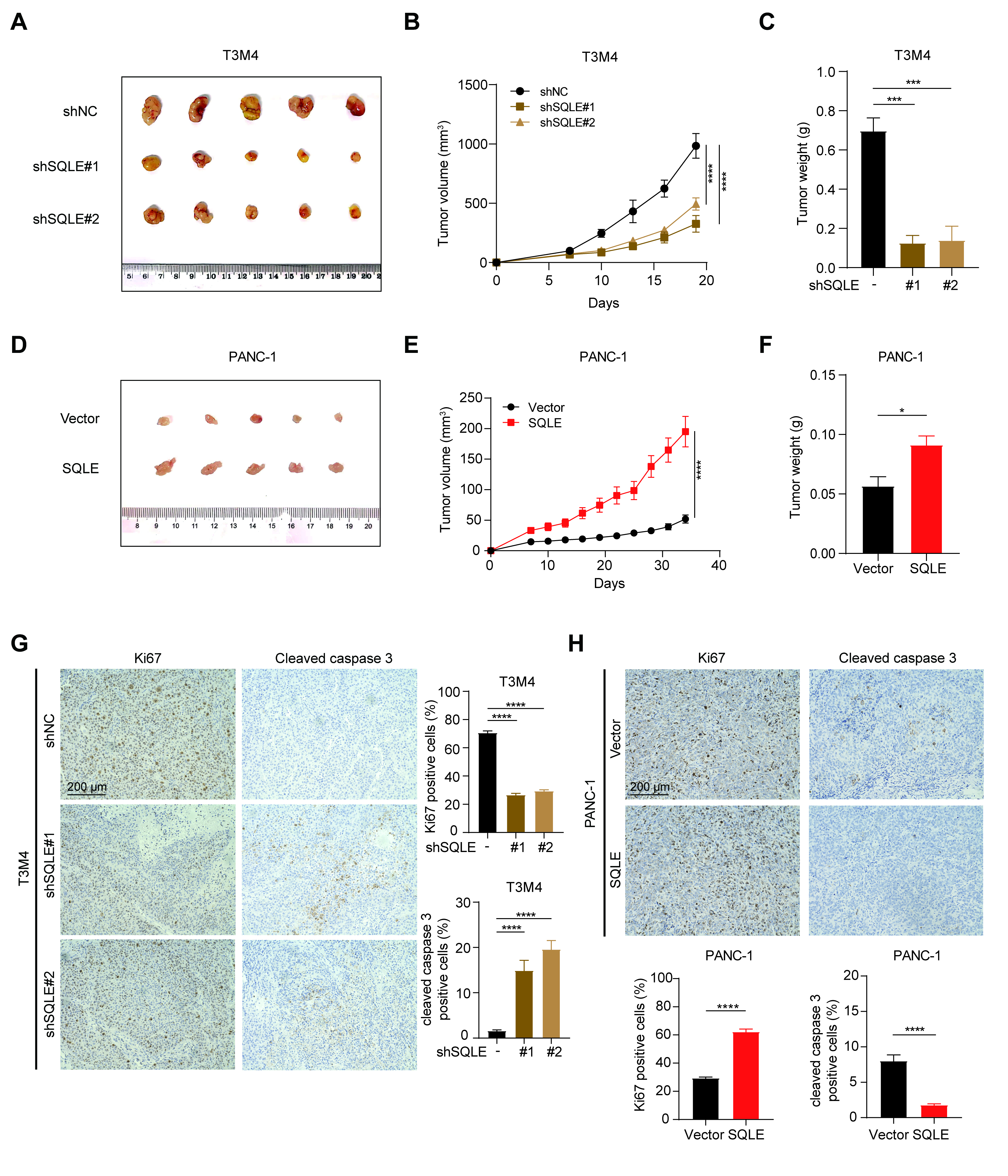

Supplement: Supplementary file 2 — Supplementary Figure S2 [file 41419_2023_5987_MOESM2_ESM.tif]

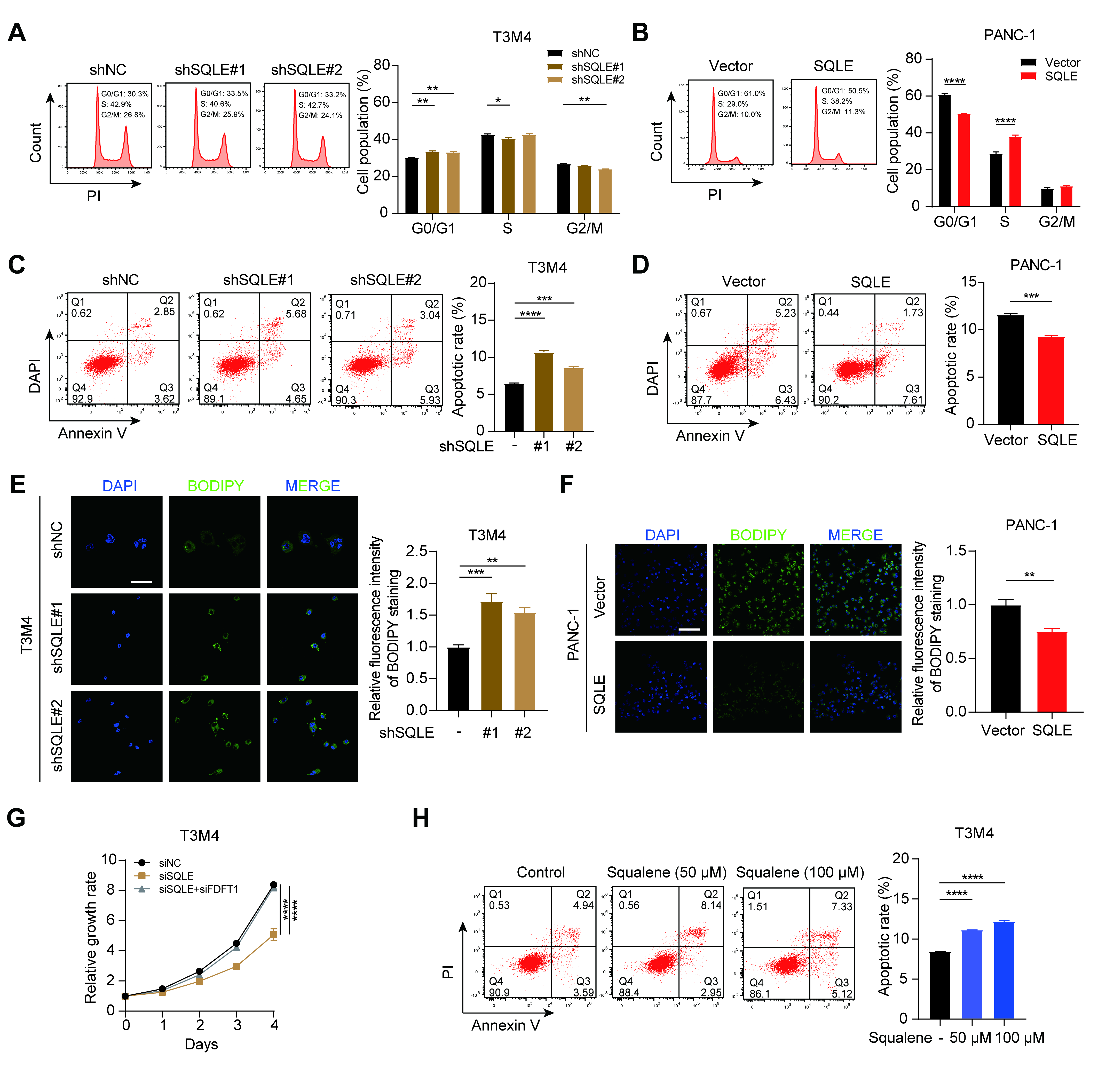

Supplement: Supplementary file 3 — Supplementary Figure S3 [file 41419_2023_5987_MOESM3_ESM.tif]

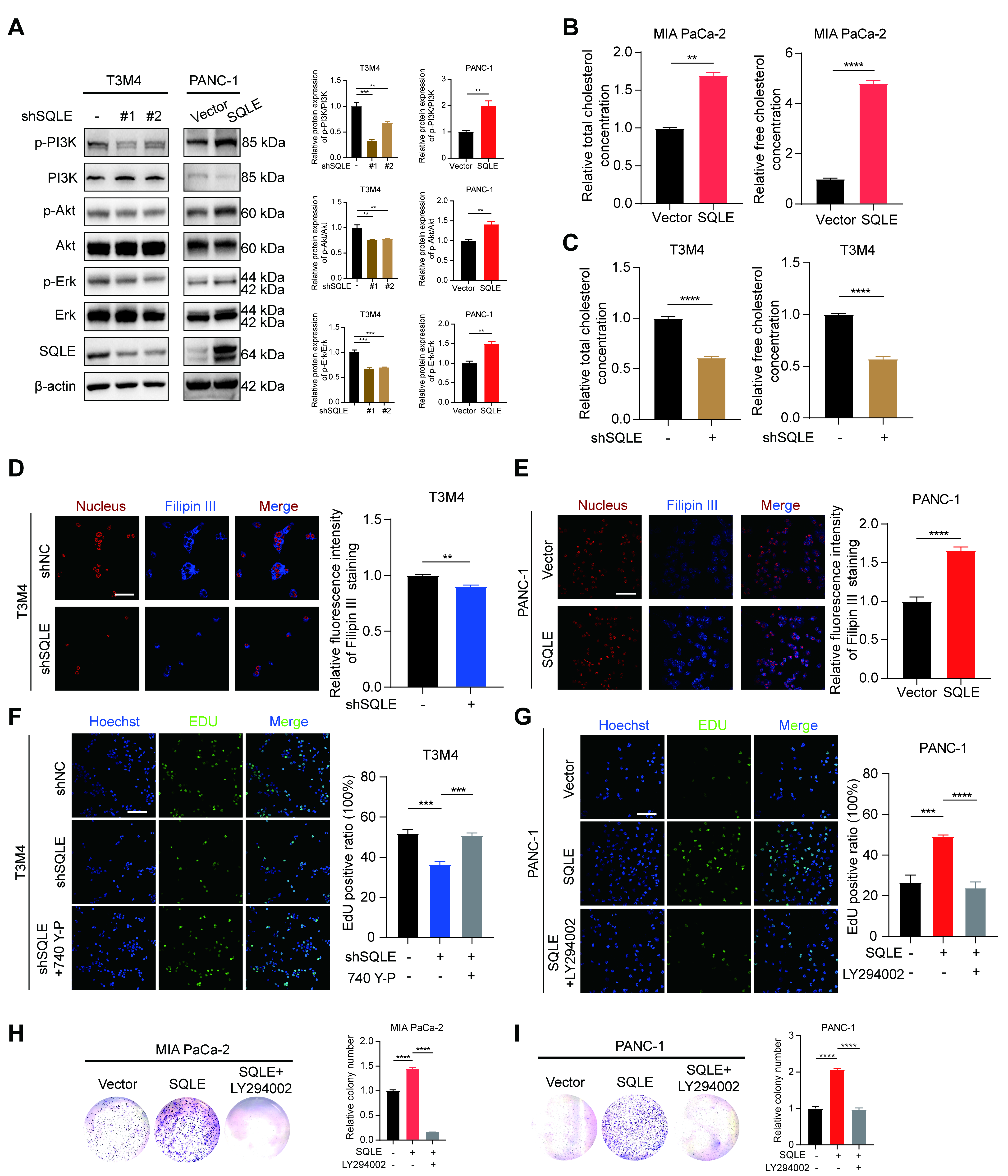

Supplement: Supplementary file 4 — Supplementary Figure S4 [file 41419_2023_5987_MOESM4_ESM.tif]

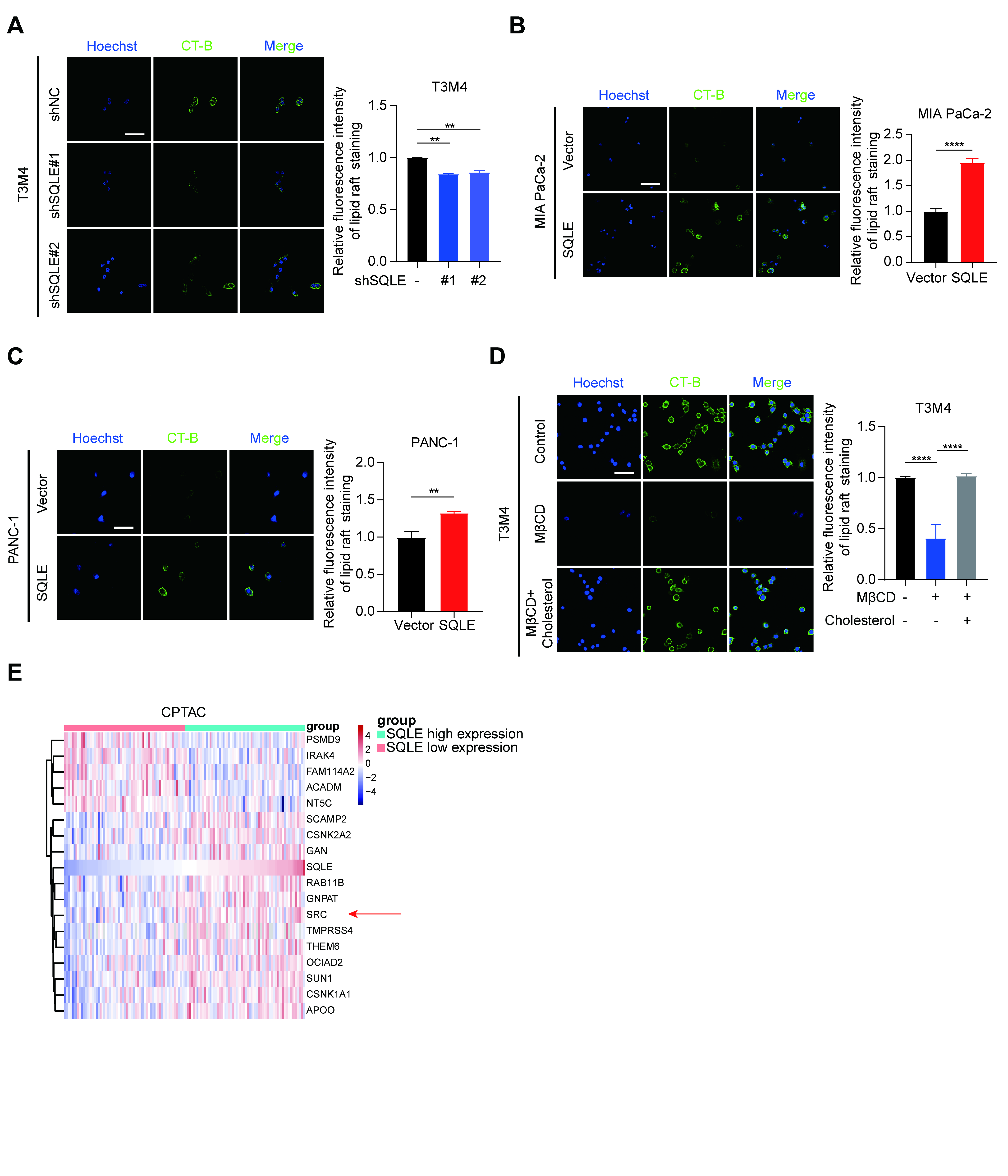

Supplement: Supplementary file 5 — Supplementary Figure S5 [file 41419_2023_5987_MOESM5_ESM.tif]

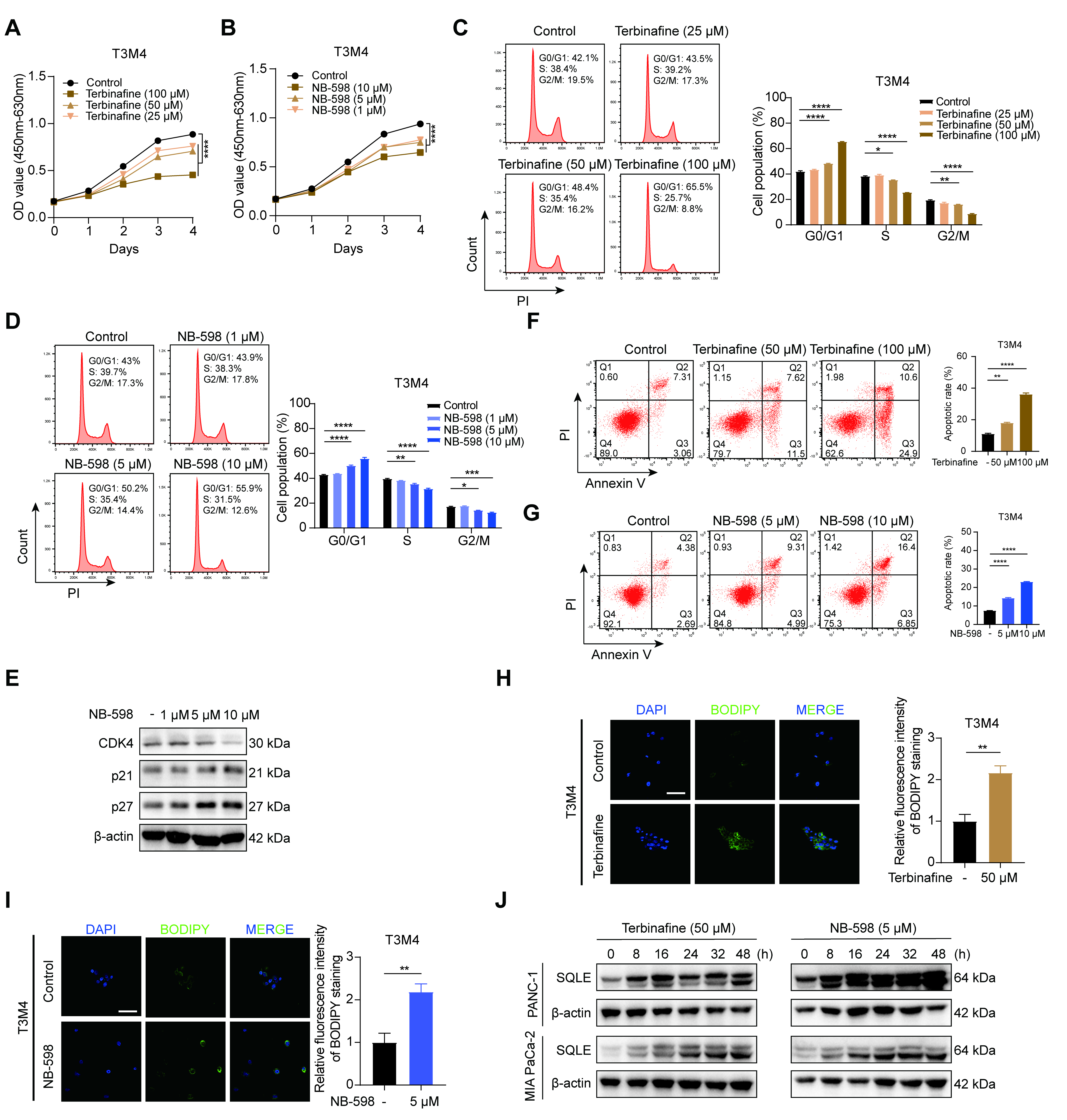

Supplement: Supplementary file 6 — Supplementary Figure S6 [file 41419_2023_5987_MOESM6_ESM.tif]

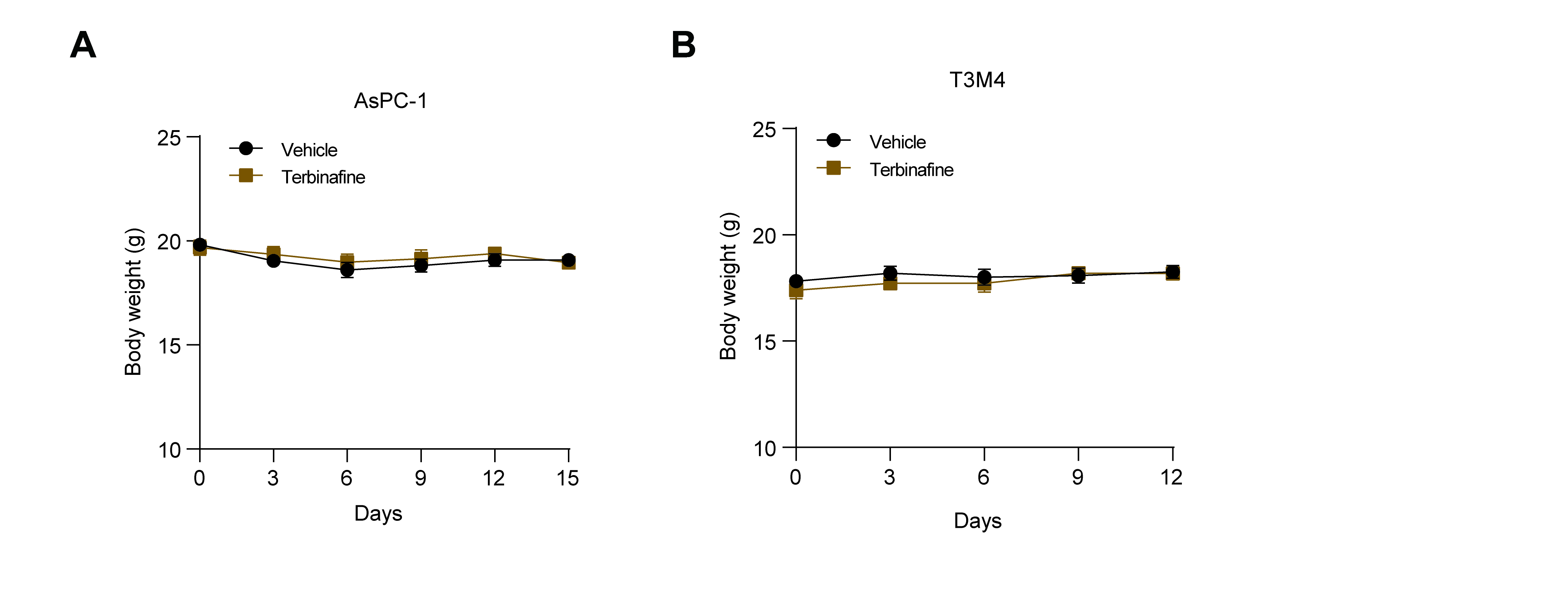

Supplement: Supplementary file 7 — Supplementary Figure S7 [file 41419_2023_5987_MOESM7_ESM.tif]
