# Supplementary material for: SQLE promotes pancreatic cancer growth by attenuating ER stress and activating lipid rafts-regulated Src/PI3K/Akt signaling pathway
Source: Cell Death Dis. 2023 Aug 4;14(8):497. doi: 10.1038/s41419-023-05987-7 (PMC10403582; doi:10.1038/s41419-023-05987-7)
Supplement: Supplementary file 8 — Supplementary Figure legends [file 41419_2023_5987_MOESM8_ESM.docx]

**Supplementary Figure legends**

**Supplementary Figure S1** The efficiency of knockdown or overexpression of SQLE in PC cell lines by real-time PCR. **A-B.** Knockdown of SQLE of T3M4 and AsPC-1 cells were confirmed by real-time PCR. **C-D.** Overexpression of SQLE of PANC-1 and MIA PaCa-2 cells were confirmed by real-time PCR. **, *P* < 0.01; ****, *P* < 0.0001.

**Supplementary Figure S2** SQLE promotes PC cell proliferation *in vivo*. **A.** Images of isolated tumors from the subcutaneous tumor nude mice model established using T3M4 cells expressing control or SQLE shRNA (n = 5 mice per group). **B-C.** Tumor volume and weight in control and SQLE knockdown T3M4 groups. **D.** Images of isolated tumors from the subcutaneous tumor nude mice model established using PANC-1 cells expressing vector or SQLE overexpression (n = 5 mice per group). **E-F.** Tumor volume and weight in vector and SQLE overexpression PANC-1 groups. **G.** Left, representative images of immunohistochemistry for Ki67 and cleaved caspase 3 staining in xenografts derived from T3M4 cells. Scale bar: 200 μm. Right, statistical analysis of Ki67 and cleaved caspase 3 positive cells. **H.** Top, representative images of immunohistochemistry for Ki67 and cleaved caspase 3 staining in xenografts derived from PANC-1 cells. Scale bar: 200 μm. Bottom, statistical analysis of Ki67 and cleaved caspase 3 positive cells. *, *P* < 0.05; ***, *P* < 0.001; ****, *P* < 0.0001.

**Supplementary Figure S3** SQLE promotes cell cycle progression and suppresses ER stress-triggered cell apoptosis. **A.** Cell cycle analysis of T3M4 cells expressing control or SQLE shRNA. **B.** Cell cycle analysis of PANC-1 cells expressing vector or SQLE overexpression. **C.** Cell apoptosis analysis of T3M4 cells expressing control or SQLE shRNA. **D.** Cell apoptosis analysis of PANC-1 cells expressing vector or SQLE overexpression. **E-F.** Detection of formation of lipid droplets using BODIPY 493/503 staining in T3M4 cells expressing control and SQLE shRNA or in PANC-1 cells expressing vector and SQLE overexpression. Left panel, representative images of BODIPY staining. Right panel, statistical analysis of relative fluorescence intensity of BODIPY staining. Scale bar: 100 μm. **G.** Cell growth curves of T3M4 with ± SQLE knockdown and ± FDFT1 knockdown. **H.** Cell apoptosis analysis of T3M4 cells treated with 0, 50, and 100 μM squalene for 48h. Left panel, representative images of flow cytometry. Right panel, statistical analysis of apoptotic rate of treated cells. *, *P* < 0.05; **, *P* < 0.01; ***, *P* < 0.001; ****, *P* < 0.0001.

**Supplementary Figure S4** SQLE promotes PC cell growth by activating downstream PI3K/Akt and MAPK signaling pathways. **A.** Western blotting analysis showing the expression of PI3K, p-PI3K, Akt, p-Akt, Erk, and p-Erk after SQLE knockdown in T3M4 cells and overexpression in PANC-1 cells. **B.** The total/free cholesterol levels in MIA PaCa-2 cells expressing vector or SQLE overexpression were determined using the cholesterol quantification assay kit. **C.** The total/free cholesterol levels in T3M4 cells expressing control or SQLE shRNA were determined using the cholesterol quantification assay kit. **D.** Filipin III staining showing the cellular free cholesterol content in T3M4 cells expressing control or SQLE shRNA. Left panel, representative images of Filipin III staining. Right panel, statistical analysis of relative fluorescence intensity of Filipin III staining. Scale bar: 100 μm. **E.** Filipin III staining showing the cellular free cholesterol content in PANC-1 cells expressing vector or SQLE overexpression. Left panel, representative images of Filipin III staining. Right panel, statistical analysis of relative fluorescence intensity of Filipin III staining. Scale bar: 100 μm. **F.** Representative images of EdU staining (left panel) and EdU positive ratio (right panel) of T3M4 cells with ± SQLE knockdown and with/without 10 μM 740 Y-P treatment. Scale bar: 100 μm. **G.** Representative images of EdU staining (left panel) and EdU positive ratio (right panel) of PANC-1 cells with ± SQLE overexpression and with/without 10 μM LY294002 treatment. Scale bar: 100 μm. **H.** Colony formation of MIA PaCa-2 cells with ± SQLE overexpression and with/without 10 μM LY294002 treatment. **I.** Colony formation of PANC-1 cells with ± SQLE overexpression and with/without 10 μM LY294002 treatment. **, *P* < 0.01; ***, *P* < 0.001; ****, *P* < 0.0001.

**Supplementary Figure S5** SQLE regulates the Src/PI3K/Akt cascade via cholesterol-dependent lipid rafts. **A.** CT-B staining showing the contents of lipid rafts in T3M4 cells expressing control or SQLE shRNA. Left panel, representative images of CT-B staining. Right panel, statistical analysis of relative fluorescence intensity of CT-B staining. Scale bar: 100 μm. **B.** CT-B staining showing the contents of lipid rafts in MIA PaCa-2 cells expressing vector or SQLE overexpression. Left panel, representative images of CT-B staining. Right panel, statistical analysis of relative fluorescence intensity of CT-B staining. Scale bar: 100 μm. **C.** CT-B staining showing the contents of lipid rafts in PANC-1 cells expressing vector or SQLE overexpression. Left panel, representative images of CT-B staining. Right panel, statistical analysis of relative fluorescence intensity of CT-B staining. Scale bar: 100 μm. **D.** CT-B staining showing the contents of lipid rafts in T3M4 cells after treatment with 10mM MβCD for 30 min or 10mM MβCD for 30 min + 10 μM cholesterol overnight. Left panel, representative images of CT-B staining. Right panel, statistical analysis of relative fluorescence intensity of CT-B staining. Scale bar: 100 μm. **E.** The heatmap showing the differential protein expression between SQLE high expression and SQLE low expression group through analyzing CPTAC dataset. The red arrow indicated Src. **, *P* < 0.01. ****, *P* < 0.0001.

**Supplementary Figure S6** SQLE inhibitors terbinafine and NB-598 suppressed proliferation, induced cell cycle arrest and activated ER stress-triggered apoptosis of PC cells in vitro. **A.** Cell growth curves of T3M4 cells treated with 0, 25, 50, and 100 μM terbinafine for 4d. **B.** Cell growth curves of T3M4 cells treated with 0, 1, 5, and 10 μM NB-598 for 4d. **C.** Cell cycle analysis of T3M4 cells treated with 0, 25, 50, and 100 μM terbinafine for 48h. **D.** Cell cycle analysis of T3M4 cells treated with 0, 1, 5, and 10 μM NB-598 for 48h. **E.** Western blotting images showing the expression of cell cycle-related proteins CDK4, p21, and p27 in AsPC-1 cells treated with 0, 1, 5, and 10 μM NB-598 for 48h. **F.** Cell apoptosis analysis of T3M4 cells treated with 0, 50, and 100 μM terbinafine for 48h. **G.** Cell apoptosis analysis of T3M4 cells treated with 0, 5, and 10 μM NB-598 for 48h. **H.** Detection of formation of lipid droplets using BODIPY 493/503 staining in T3M4 cells treated with 0 and 50 μM terbinafine for 48h. Left panel, representative images of BODIPY staining. Right panel, statistical analysis of relative fluorescence intensity of BODIPY staining. Scale bar: 100 μm. **I.** Detection of formation of lipid droplets using BODIPY 493/503 staining in T3M4 cells treated with 0 and 5 μM NB-598 for 48h. Left panel, representative images of BODIPY staining. Right panel, statistical analysis of relative fluorescence intensity of BODIPY staining. Scale bar: 100 μm. **J.** Western blotting images showing the expression of SQLE in PANC-1 and MIA PaCa-2 cells treated with 50 μM terbinafine (left panel) and 5 μM NB-598 (right panel) for 0, 8, 16, 24, 32, and 48 h. *, *P* < 0.05; **, *P* < 0.01; ***, *P* < 0.001; ****, *P* < 0.0001.

**Supplementary Figure S7** SQLE inhibitor attenuates PC tumor growth. **A-B.** Curves showing body weight changes of mice bearing AsPC-1 and T3M4 xenografts with vehicle or terbinafine (orally, 80 mg/kg).
