# Supplementary material for: SQLE promotes pancreatic cancer growth by attenuating ER stress and activating lipid rafts-regulated Src/PI3K/Akt signaling pathway
Source: Cell Death Dis. 2023 Aug 4;14(8):497. doi: 10.1038/s41419-023-05987-7 (PMC10403582; doi:10.1038/s41419-023-05987-7)
Supplement: Supplementary file 9 — Supplementary Table S1 [file 41419_2023_5987_MOESM9_ESM.docx]

**Supplementary Table S1** Details of antibodies

| **Antibodies** | **Company** | **Cat #** | **Dilution** |
| --- | --- | --- | --- |
| SQLE | Proteintech | 12544-1-AP | 1:1000 |
| GRP78 | Cell Signaling Technology | 3177 | 1:1000 |
| eIF2α | Cell Signaling Technology | 5324 | 1:1000 |
| Phospho-eIF2α (Ser51) | Cell Signaling Technology | 3398 | 1:1000 |
| ATF4 | Proteintech | 10835-1-AP | 1:1000 |
| CHOP | Proteintech | 15204-1-AP | 1:1000 |
| FDFT1 | Proteintech | 13128-1-AP | 1:5000 |
| Caspase-3 | Cell Signaling Technology | 14220 | 1:1000 |
| Cleaved Caspase-3 (Asp175) | Cell Signaling Technology | 9661 | 1:500 |
| PI3 Kinase p85 | Cell Signaling Technology | 4257 | 1:1000 |
| Phospho-PI3 Kinase p85 (Tyr458)/p55 (Tyr199) | Cell Signaling Technology | 4228 | 1:1000 |
| Akt (pan) | Cell Signaling Technology | 4691 | 1:1000 |
| Phospho-Akt (Ser473) | Cell Signaling Technology | 4060 | 1:2000 |
| p44/42 MAPK (Erk1/2) | Cell Signaling Technology | 4695 | 1:1000 |
| Phospho-p44/42 MAPK (Erk1/2) (Thr202/Tyr204) | Cell Signaling Technology | 4370 | 1:2000 |
| Src | Cell Signaling Technology | 2109 | 1:1000 |
| Phospho-Src Family (Tyr416) | Cell Signaling Technology | 6943 | 1:1000 |
| CDK4 | Cell Signaling Technology | 12790 | 1:1000 |
| p21 | Cell Signaling Technology | 2947 | 1:1000 |
| p27 | Proteintech | 25614-1-AP | 1:1000 |
| β-actin | Proteintech | 66009-1-Ig | 1:20000 |
| HRP-conjugated Anti-Mouse IgG | Proteintech | SA00001-1 | 1:5000 |
| HRP-conjugated Anti-Rabbit IgG | Proteintech | SA00001-2 | 1:5000 |
