# Supplementary material for: SQLE promotes pancreatic cancer growth by attenuating ER stress and activating lipid rafts-regulated Src/PI3K/Akt signaling pathway
Source: Cell Death Dis. 2023 Aug 4;14(8):497. doi: 10.1038/s41419-023-05987-7 (PMC10403582; doi:10.1038/s41419-023-05987-7)
Supplement: Supplementary file 10 — Supplementary Table S2 [file 41419_2023_5987_MOESM10_ESM.docx]

**Supplementary Table S2** The full list of gene set GOBP_REGULATION_OF_CHOLESTEROL_METABOLIC_PROCESS

| ABCG1 | EPHX2 | LDLRAP1 | NR1H4 |
| --- | --- | --- | --- |
| ACACA | ERLIN1 | LPCAT3 | PMVK |
| ACACB | ERLIN2 | LSS | PRKAA1 |
| ACADL | FASN | MBTPS1 | RAN |
| ACADVL | FDFT1 | MBTPS2 | SC5D |
| APOB | FDPS | MIR182 | SCAP |
| APOE | FGF1 | MIR185 | SCD |
| ARV1 | FMO5 | MIR30C1 | SEC14L2 |
| CES1 | GGPS1 | MIR548P | SERPINA12 |
| CYP51A1 | GNB3 | MIR96 | SOD1 |
| CYP7A1 | GPAM | MVD | SP1 |
| DGAT2 | HMGCR | MVK | SQLE |
| DGKQ | HMGCS1 | NFYA | SREBF1 |
| DHCR7 | IDI1 | NFYB | SREBF2 |
| ELOVL6 | KPNB1 | NFYC | TM7SF2 |
| TTC39B |  |  |  |
