# Supplementary material for: SQLE promotes pancreatic cancer growth by attenuating ER stress and activating lipid rafts-regulated Src/PI3K/Akt signaling pathway
Source: Cell Death Dis. 2023 Aug 4;14(8):497. doi: 10.1038/s41419-023-05987-7 (PMC10403582; doi:10.1038/s41419-023-05987-7)
Supplement: Supplementary file 11 — Supplementary Table S3 [file 41419_2023_5987_MOESM11_ESM.docx]

**Supplementary Table S3** Correlation between SQLE expression and clinicopathological characteristics in patients with PC

| **Clinicopathological variables** | **Total** | **SQLE low** | **SQLE high** | **P-value** |
| --- | --- | --- | --- | --- |
|  | 83 | 40 | 43 |  |
| **Gender** |  |  |  | 0.050 |
| Female | 34 | 12 | 22 |  |
| Male | 49 | 28 | 21 |  |
| **Age** |  |  |  | 0.487 |
| < 65 | 61 | 28 | 33 |  |
| ≥ 65 | 22 | 12 | 10 |  |
| **Tumor grade** |  |  |  | 0.693 |
| Well | 2 | 1 | 1 |  |
| Moderately | 48 | 25 | 23 |  |
| Poorly | 33 | 14 | 19 |  |
| **Stage** |  |  |  | <0.0001 |
| I-II | 49 | 35 | 14 |  |
| III-IV | 34 | 5 | 29 |  |
| **T stage** |  |  |  | 0.062 |
| T1 - T2 | 41 | 24 | 17 |  |
| T3 - T4 | 42 | 16 | 26 |  |
| **N stage** |  |  |  | 0.038 |
| N0 | 30 | 19 | 11 |  |
| N1 - N2 | 53 | 21 | 32 |  |
| **M stage** |  |  |  | <0.0001 |
| M0 | 61 | 40 | 21 |  |
| M1 | 22 | 0 | 22 |  |
