# Supplementary material for: SQLE promotes pancreatic cancer growth by attenuating ER stress and activating lipid rafts-regulated Src/PI3K/Akt signaling pathway
Source: Cell Death Dis. 2023 Aug 4;14(8):497. doi: 10.1038/s41419-023-05987-7 (PMC10403582; doi:10.1038/s41419-023-05987-7)
Supplement: Supplementary file 12 — Original western blots [file 41419_2023_5987_MOESM12_ESM.pptx]

## Slide 1
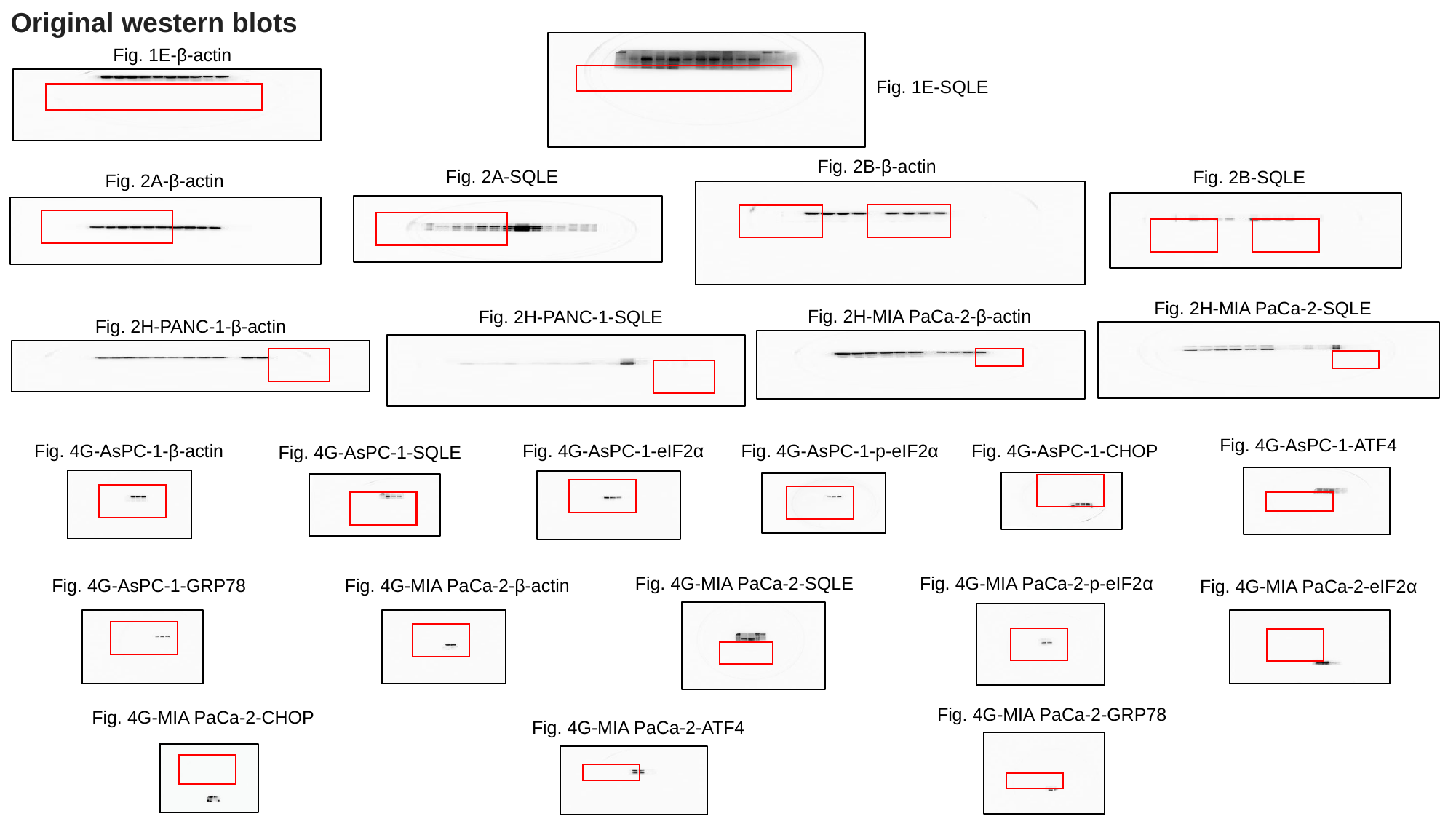

Original western blots
Fig. 1E-β-actin
Fig. 1E-SQLE
Fig. 2B-β-actin
Fig. 2A-SQLE
Fig. 2B-SQLE
Fig. 2A-β-actin
Fig. 2H-MIA PaCa-2-SQLE
Fig. 2H-MIA PaCa-2-β-actin
Fig. 2H-PANC-1-SQLE
Fig. 2H-PANC-1-β-actin
Fig. 4G-AsPC-1-ATF4
Fig. 4G-AsPC-1-p-eIF2α
Fig. 4G-AsPC-1-β-actin
Fig. 4G-AsPC-1-eIF2α
Fig. 4G-AsPC-1-CHOP
Fig. 4G-AsPC-1-SQLE
Fig. 4G-MIA PaCa-2-SQLE
Fig. 4G-MIA PaCa-2-p-eIF2α
Fig. 4G-AsPC-1-GRP78
Fig. 4G-MIA PaCa-2-β-actin
Fig. 4G-MIA PaCa-2-eIF2α
Fig. 4G-MIA PaCa-2-GRP78
Fig. 4G-MIA PaCa-2-CHOP
Fig. 4G-MIA PaCa-2-ATF4

## Slide 2
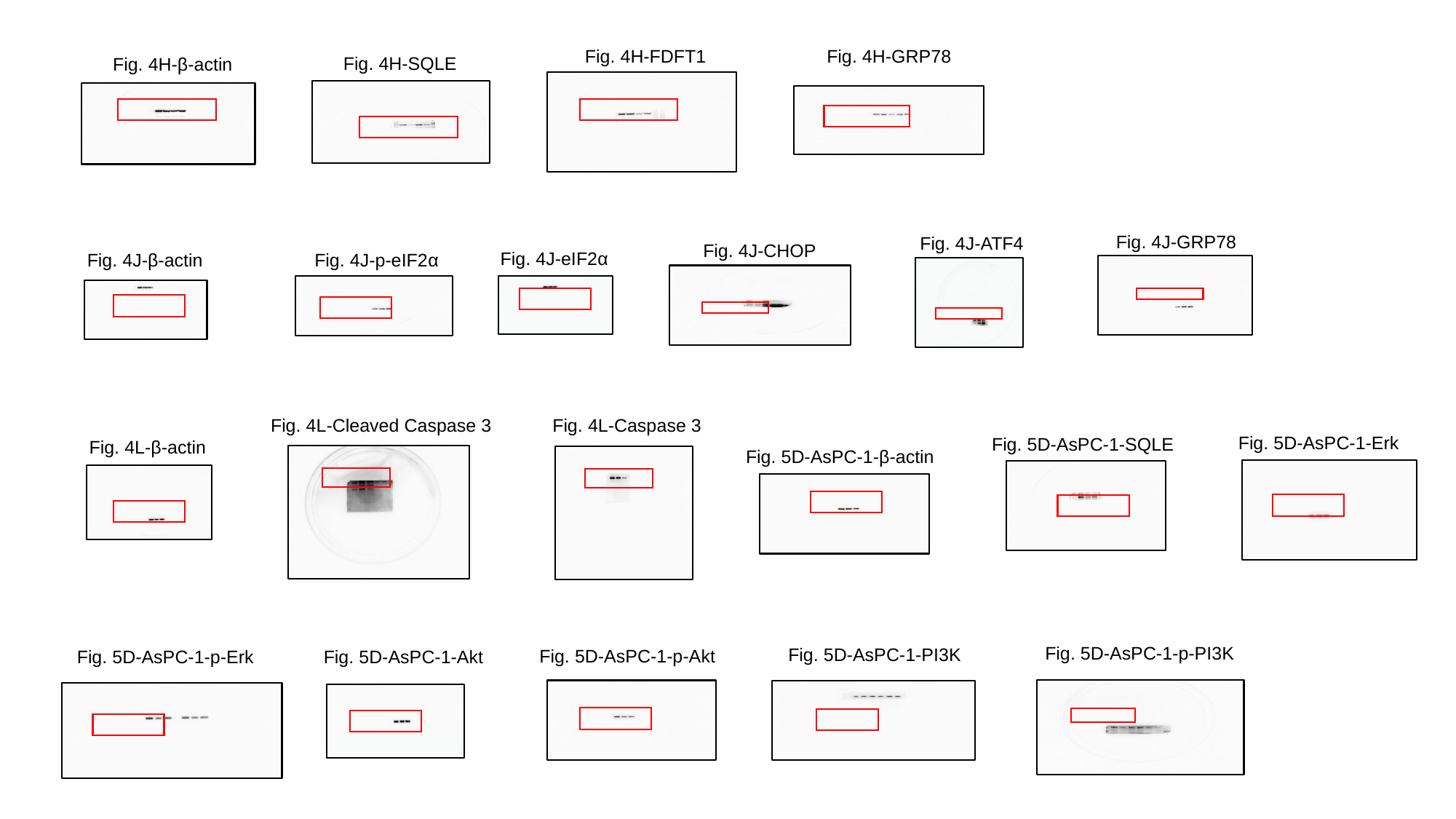

Fig. 4H-GRP78
Fig. 4H-FDFT1
Fig. 4H-SQLE
Fig. 4H-β-actin
Fig. 4J-GRP78
Fig. 4J-ATF4
Fig. 4J-CHOP
Fig. 4J-eIF2α
Fig. 4J-β-actin
Fig. 4J-p-eIF2α
Fig. 4L-Cleaved Caspase 3
Fig. 4L-Caspase 3
Fig. 5D-AsPC-1-Erk
Fig. 5D-AsPC-1-SQLE
Fig. 4L-β-actin
Fig. 5D-AsPC-1-β-actin
Fig. 5D-AsPC-1-p-PI3K
Fig. 5D-AsPC-1-PI3K
Fig. 5D-AsPC-1-p-Akt
Fig. 5D-AsPC-1-Akt
Fig. 5D-AsPC-1-p-Erk

## Slide 3
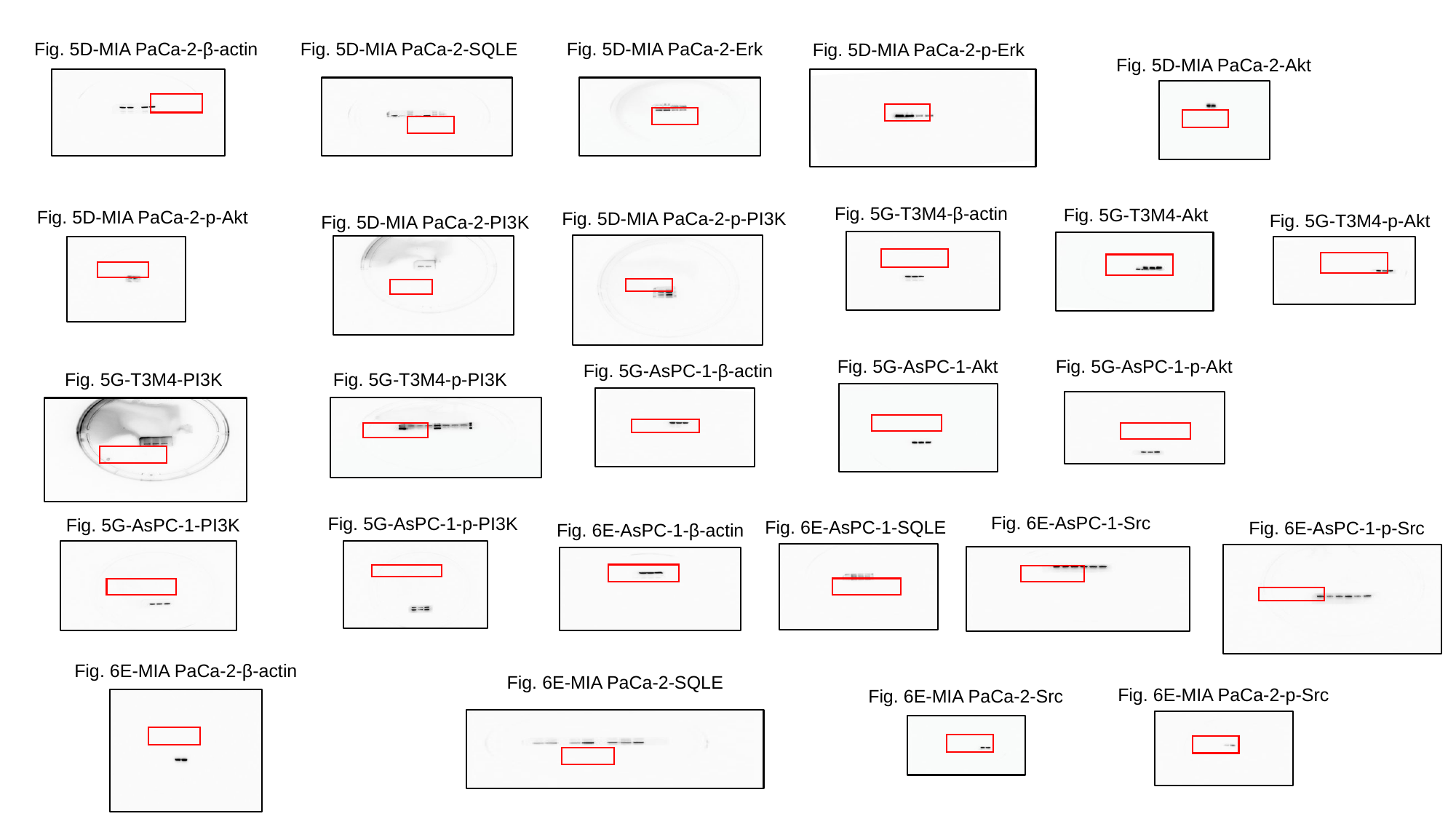

Fig. 5D-MIA PaCa-2-β-actin
Fig. 5D-MIA PaCa-2-SQLE
Fig. 5D-MIA PaCa-2-Erk
Fig. 5D-MIA PaCa-2-p-Erk
Fig. 5D-MIA PaCa-2-Akt
Fig. 5G-T3M4-β-actin
Fig. 5G-T3M4-Akt
Fig. 5D-MIA PaCa-2-p-Akt
Fig. 5D-MIA PaCa-2-p-PI3K
Fig. 5G-T3M4-p-Akt
Fig. 5D-MIA PaCa-2-PI3K
Fig. 5G-AsPC-1-p-Akt
Fig. 5G-AsPC-1-Akt
Fig. 5G-AsPC-1-β-actin
Fig. 5G-T3M4-p-PI3K
Fig. 5G-T3M4-PI3K
Fig. 6E-AsPC-1-Src
Fig. 5G-AsPC-1-p-PI3K
Fig. 5G-AsPC-1-PI3K
Fig. 6E-AsPC-1-SQLE
Fig. 6E-AsPC-1-p-Src
Fig. 6E-AsPC-1-β-actin
Fig. 6E-MIA PaCa-2-β-actin
Fig. 6E-MIA PaCa-2-SQLE
Fig. 6E-MIA PaCa-2-p-Src
Fig. 6E-MIA PaCa-2-Src

## Slide 4
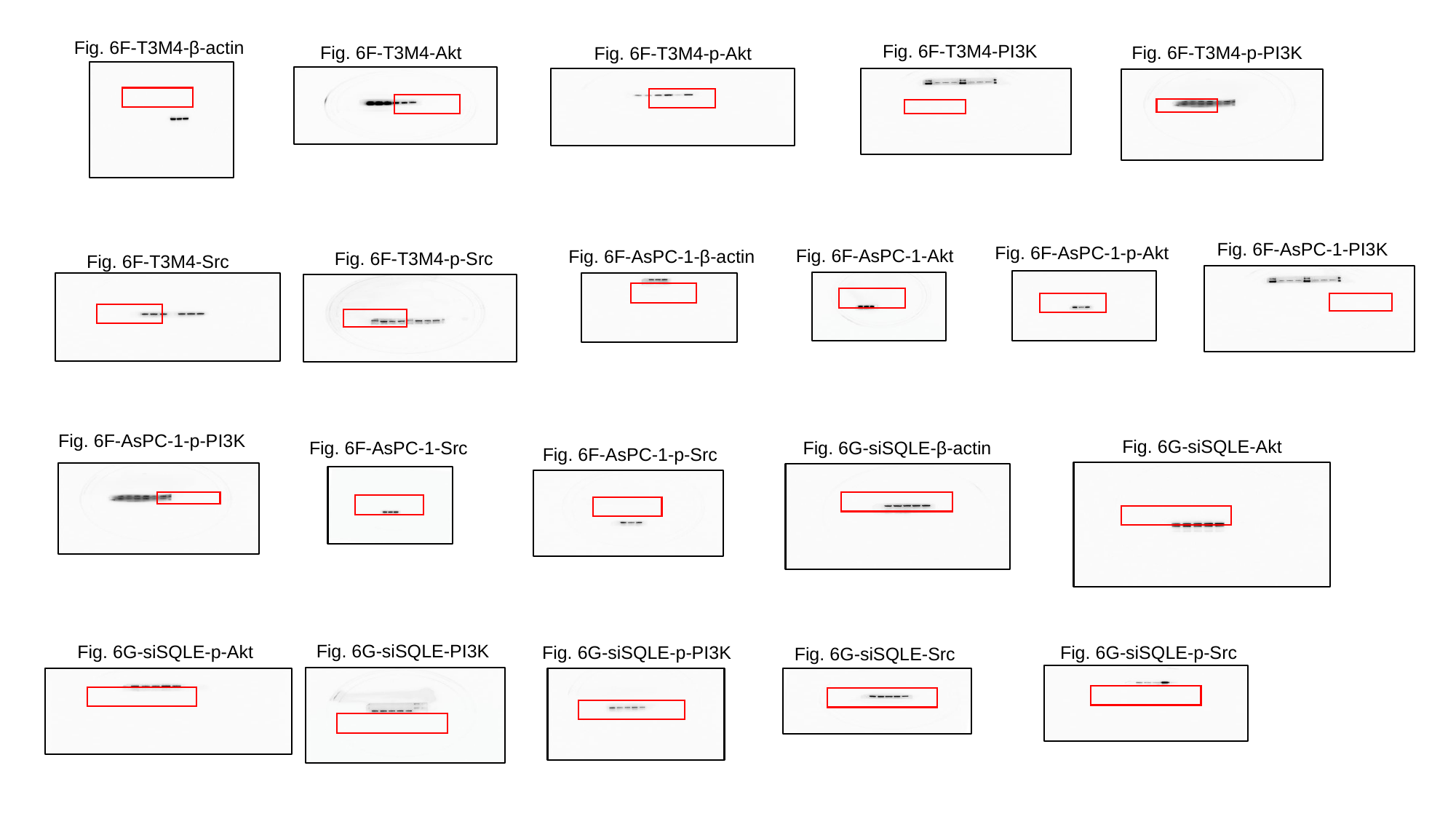

Fig. 6F-T3M4-β-actin
Fig. 6F-T3M4-PI3K
Fig. 6F-T3M4-Akt
Fig. 6F-T3M4-p-PI3K
Fig. 6F-T3M4-p-Akt
Fig. 6F-AsPC-1-PI3K
Fig. 6F-AsPC-1-p-Akt
Fig. 6F-AsPC-1-Akt
Fig. 6F-AsPC-1-β-actin
Fig. 6F-T3M4-p-Src
Fig. 6F-T3M4-Src
Fig. 6F-AsPC-1-p-PI3K
Fig. 6G-siSQLE-Akt
Fig. 6F-AsPC-1-Src
Fig. 6G-siSQLE-β-actin
Fig. 6F-AsPC-1-p-Src
Fig. 6G-siSQLE-PI3K
Fig. 6G-siSQLE-p-Akt
Fig. 6G-siSQLE-p-PI3K
Fig. 6G-siSQLE-p-Src
Fig. 6G-siSQLE-Src

## Slide 5
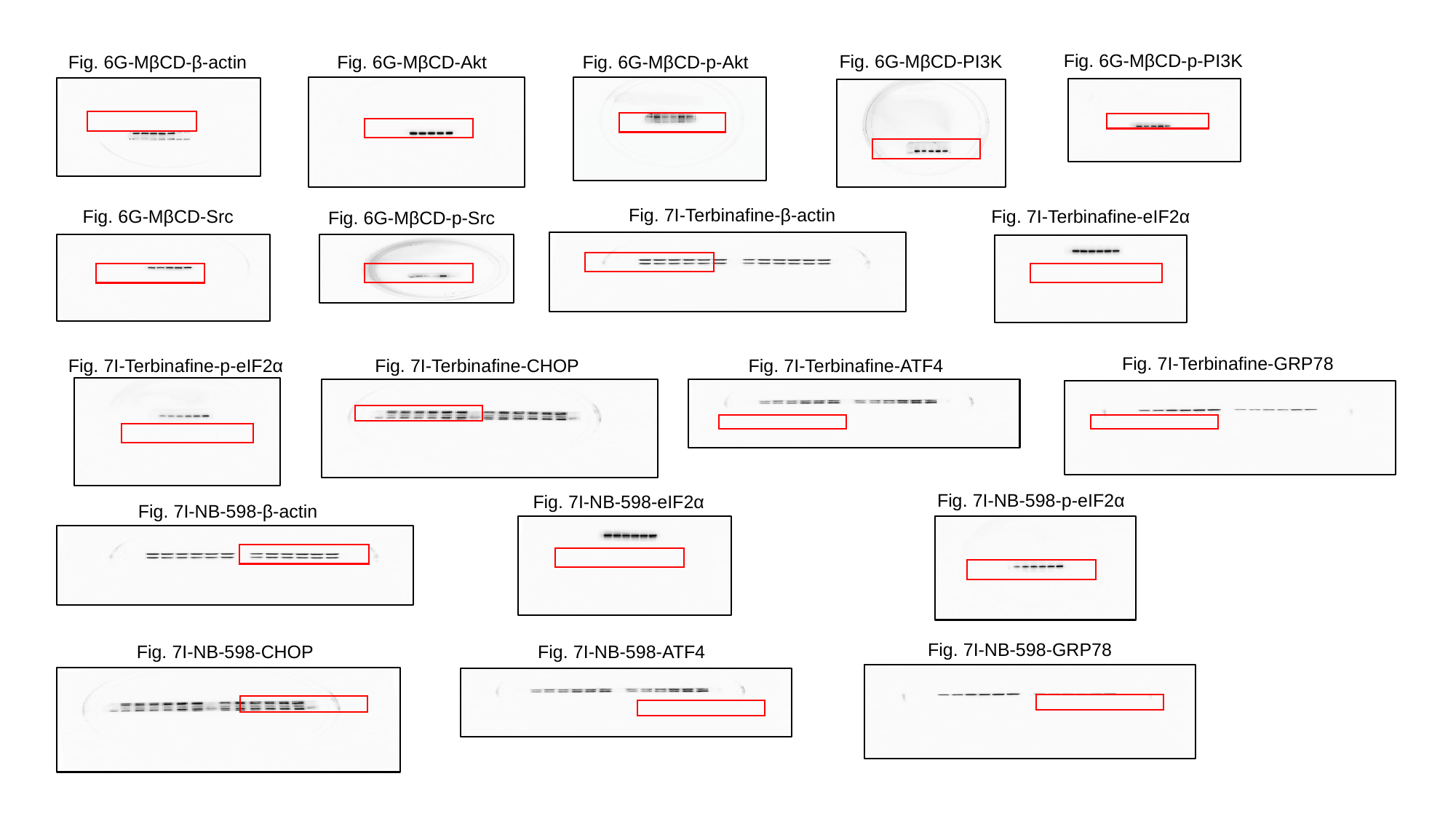

Fig. 6G-MβCD-p-PI3K
Fig. 6G-MβCD-PI3K
Fig. 6G-MβCD-Akt
Fig. 6G-MβCD-β-actin
Fig. 6G-MβCD-p-Akt
Fig. 7I-Terbinafine-β-actin
Fig. 7I-Terbinafine-eIF2α
Fig. 6G-MβCD-Src
Fig. 6G-MβCD-p-Src
Fig. 7I-Terbinafine-GRP78
Fig. 7I-Terbinafine-ATF4
Fig. 7I-Terbinafine-p-eIF2α
Fig. 7I-Terbinafine-CHOP
Fig. 7I-NB-598-p-eIF2α
Fig. 7I-NB-598-eIF2α
Fig. 7I-NB-598-β-actin
Fig. 7I-NB-598-GRP78
Fig. 7I-NB-598-ATF4
Fig. 7I-NB-598-CHOP

## Slide 6
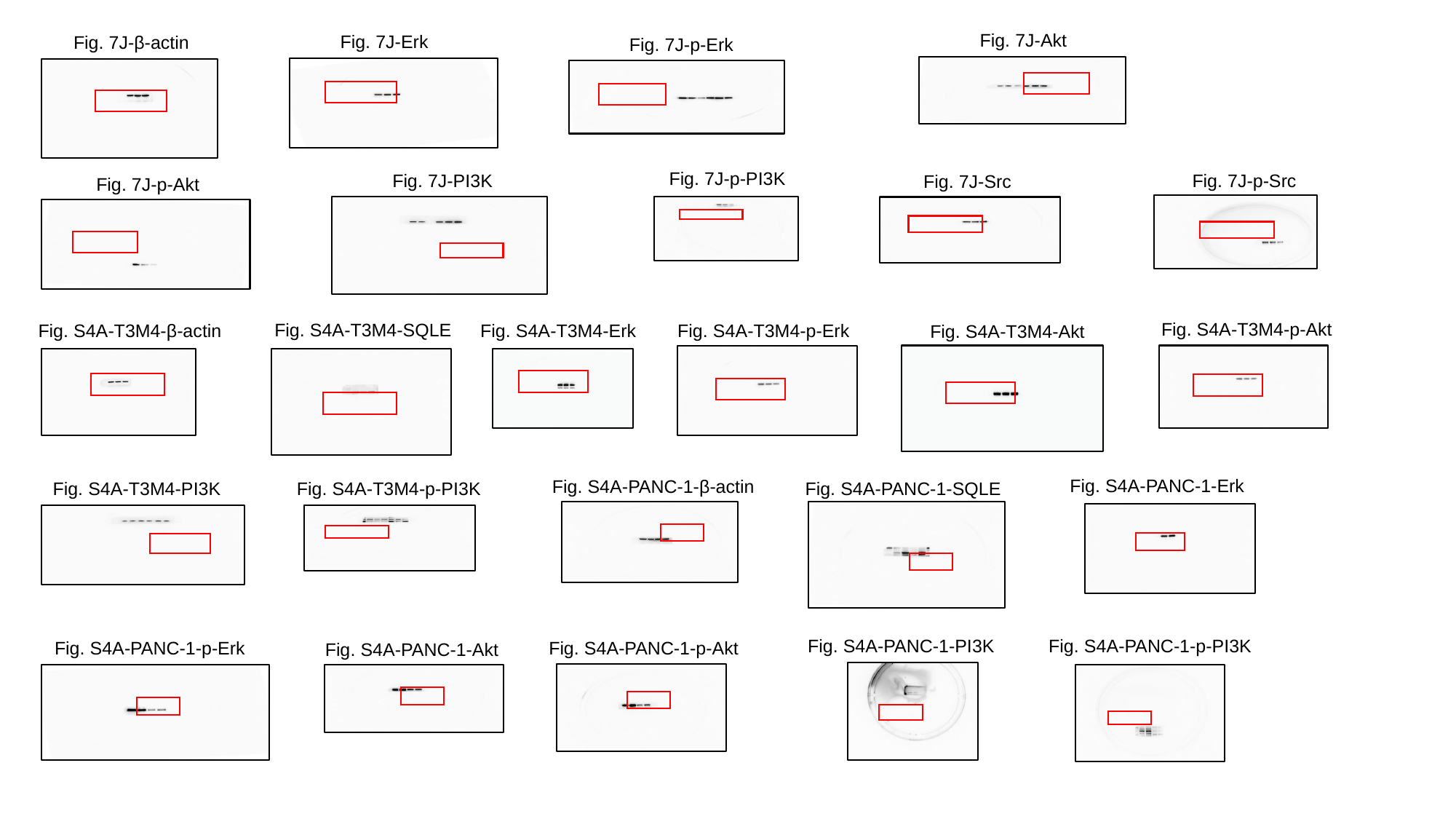

Fig. 7J-Akt
Fig. 7J-Erk
Fig. 7J-β-actin
Fig. 7J-p-Erk
Fig. 7J-p-PI3K
Fig. 7J-PI3K
Fig. 7J-p-Src
Fig. 7J-Src
Fig. 7J-p-Akt
Fig. S4A-T3M4-p-Akt
Fig. S4A-T3M4-SQLE
Fig. S4A-T3M4-p-Erk
Fig. S4A-T3M4-Erk
Fig. S4A-T3M4-β-actin
Fig. S4A-T3M4-Akt
Fig. S4A-PANC-1-Erk
Fig. S4A-PANC-1-β-actin
Fig. S4A-T3M4-p-PI3K
Fig. S4A-PANC-1-SQLE
Fig. S4A-T3M4-PI3K
Fig. S4A-PANC-1-p-PI3K
Fig. S4A-PANC-1-PI3K
Fig. S4A-PANC-1-p-Akt
Fig. S4A-PANC-1-p-Erk
Fig. S4A-PANC-1-Akt

## Slide 7
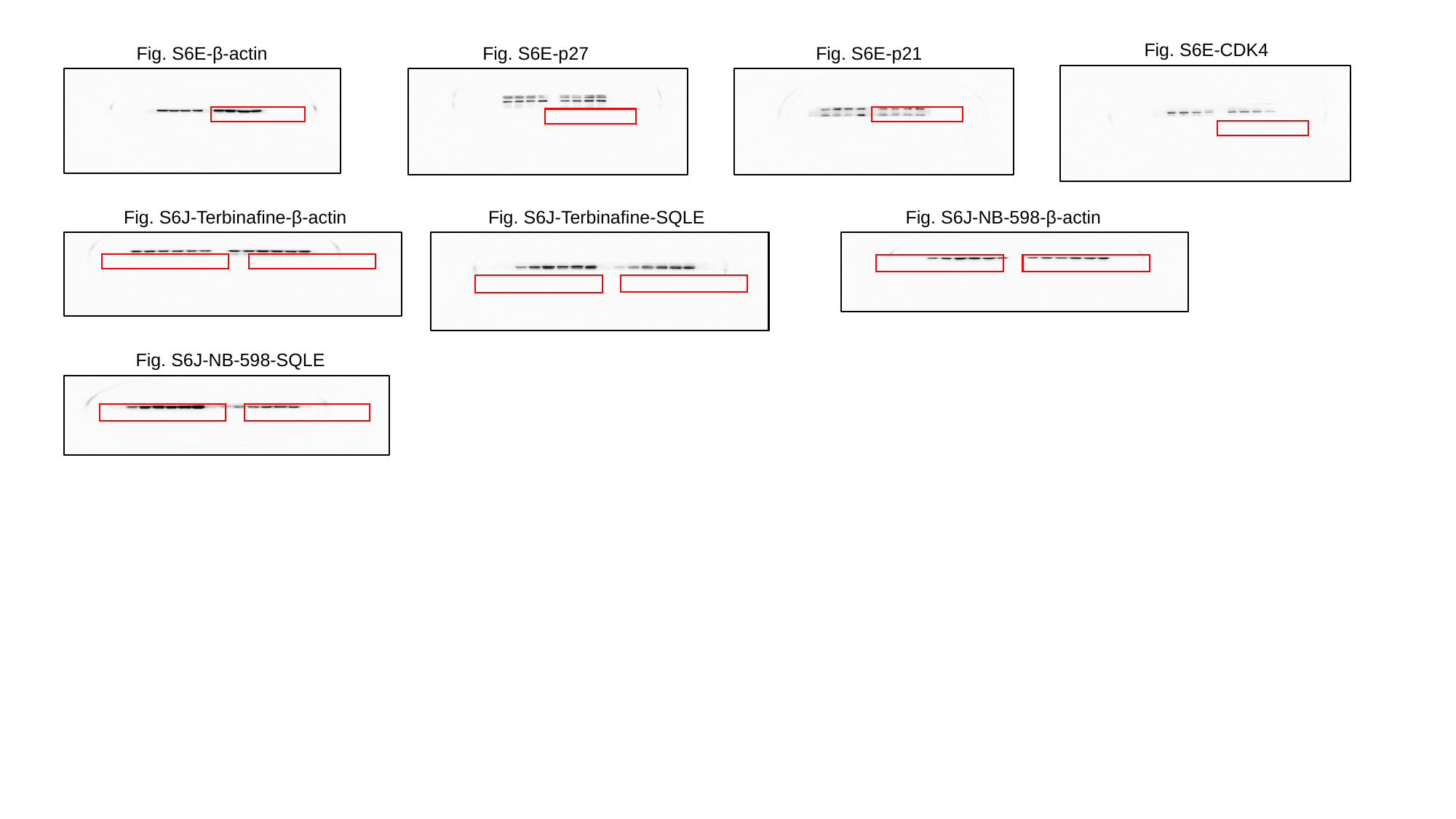

Fig. S6E-CDK4
Fig. S6E-p27
Fig. S6E-p21
Fig. S6E-β-actin
Fig. S6J-Terbinafine-β-actin
Fig. S6J-Terbinafine-SQLE
Fig. S6J-NB-598-β-actin
Fig. S6J-NB-598-SQLE
